# Exploring early Acheulian technological decision-making: A controlled experimental approach to raw material selection for percussive artifacts in Melka Wakena, Ethiopia

**DOI:** 10.1371/journal.pone.0314039

**Published:** 2025-01-09

**Authors:** Eduardo Paixão, Tegenu Gossa, Walter Gneisinger, João Marreiros, Sören Tholen, Ivan Calandra, Erella Hovers

**Affiliations:** 1 ICArEHB–Interdisciplinary Center for Archaeology and Evolution of Human Behaviour, University of Algarve, Faro, Portugal; 2 Laboratory for Traceology and Controlled Experiments (TraCEr), MONREPOS -Archaeological Research Centre and Museum for Human Behavioural Evolution, LEIZA -Leibniz-Zentrum für Archäologie, Mainz, Germany; 3 Department of Prehistory, Institute of Archaeology, Hebrew University of Jerusalem, Jerusalem, Israel; 4 Department of History and Heritage Management, Arba Minch University, Arba Minch, Ethiopia; 5 Department of Prehistoric and Protohistoric Archaeology, Institute of Ancient Studies, Johannes Gutenberg University, Mainz, Germany; 6 Tectonics and Structural Geology Working Group, Institute of Geosciences, Johannes Gutenberg University, Mainz, Germany; 7 Imaging Platform At LEIZA (IMPALA), LEIZA, Germany; 8 Institute of Human Origins, Arizona State University, Tempe, AZ, United States of America; Sapienza University of Rome: Universita degli Studi di Roma La Sapienza, ITALY

## Abstract

The evolution of human behaviour is marked by key decision-making processes reflected in technological variability in the early archaeological record. As part of the technological system, differences in raw material quality directly affect the way that humans produce, design and use stone tools. The selection, procurement and use of various raw materials requires decision-making to evaluate multiple factors such as suitability to produce and design tools, but also the materials’ efficiency and durability in performing a given task. Therefore, characterizing the physical properties of various lithic raw materials is crucial for exploring changes in human interactions with their natural environment through time and space and for understanding their technological behaviour. In this paper, we present the first step in an ongoing program designed to understand the decision-making criteria involved in the use of raw materials by the early Acheulian tool-makers at the Melka Wakena (MW) site-complex, located on the Ethiopian highlands. We present the results of the first experimental step, in which we identified and measured the engineering properties of raw materials in the lithic assemblages. These data serve as an objective, quantifiable baseline for natural experiments as well as archaeological inquiries into the technological decision-making processes of early Pleistocene hominins in Africa.

## 1. Introduction

Human behaviour across time is marked by key decision-making processes involving, among other things, the acquisition of raw materials and the manner of their consequent use. The variability of raw materials across different environments impacts their suitability for manipulation and use by humans and non-human primates [1–6]. Raw material availability and physical properties are known to have affected the ways that hominins produced, designed and used stone tools. On a broader scale, the exploitation of different raw materials influenced both the ways that past populations explored the landscape and the development of technological solutions for resource exploitation [2, 7–25]. To understand strategies that early hominin populations adopted to exploit their territories, adapt, and solve daily life challenges, it is necessary to explore the physical properties of raw materials and link them with their selection and use [26, 27]. A detailed characterization of archaeological raw materials is fundamental for exploring why some raw materials were chosen over others, and whether such choices constitute evidence of an understanding that the properties of the different raw materials render them more suitable for a given task.

Attempts to characterize archaeological raw materials have followed many analytical approaches, including naked-eye qualification of visual attributes such as colour and grain size, and the use of microscopy for the characterization of the mineralogical composition [28–31]. Researchers also paid attention to the importance of the hardness of materials [32]. Some tests such as the Leeb rebound hardness and Schmidt hammer, traditionally used in industry, have been also adopted by geologists and archaeologists to measure attributes such as the hardness of materials [9, 33, 34]. Recently the use of 3D technologies allowed researchers to collect quantitative data at different scales, including data for morphometric analyses [29, 35, 36] and detailed analyses of the surfaces including the acquisition of data on surface roughness [37].

While such analyses provide post-hoc, albeit quantified, descriptions of raw materials, our understanding of the physical properties that determine the utility of such raw materials for specific functions can also be highly improved through experimentation, which is crucial for the identification and characterization of use-wear traces at different scales of analysis [28, 38, 39]. Experimental programs typically include manual experimentation, which seeks to explore the effect of various actions and contact materials on specific raw materials. Such experiments are important and useful, yet their explanatory power is constrained by limitations on the evaluation and control of predictor variables that are themselves selected for testing based on the results of the manual replications. To tackle these limitations, several studies have adopted controlled experimental approaches that use mechanical devices and robotics, the so-called second-generation experiment (*sensu* Marreiros et al. 2020). Here the main goal is to test and isolate the variables involved in the process of surface alterations that result from human action, rather than replicating the action itself. These types of experiments are characterized by high levels of variable control, standardization, and reproducibility [32, 39–45]. The use of new technologies for mechanical experiments and their combination with the traditional approaches create new possibilities for exploring aspects related to past tool use, and inferring decision-making processes that underlie such tool selection and use [39, 42–44, 46–48].

The optimal use of stone material results from complex decision-making processes, such as availability and procurement costs, on the one hand, and physical properties (often described as “quality”) on the other hand [49, 50]. However, defining the quality of raw materials in lithic industries is not straightforward. Selection criteria might prioritize ease of production i.e., raw material “knappability”, at the expense of tool functionality and use-life duration, or vice versa. The physical properties that enhance one are not necessarily beneficial for the other and may in fact be diametrically opposed [9]. In the case of unknapped percussive tools, properties of the stone that determine knappability may be less relevant.

Non-knapped tools (e.g., percussive elements) are recognized as an important component used by many primate groups, for activities such as nut-cracking [3], seed cracking, breaking rocks and tree trunks [4], or shellfish foraging [5]. Percussive technology is important for knapping stone tools as well as for other activities, such as accessing the interior of hard-shelled fruits, breaking bones for marrow extraction or accessing the brain of big animals [51]. In the archaeological record, percussive tools are among the earliest evidence for the origin of technology, as shown by the hammerstones with concentrations of impact marks found associated with archaeological horizons dated to ca. 3.3 Ma in west Turkana, Kenya [52] and with the emergence of the human predatory pattern [51], helping to access fat-, vitamin- and protein-rich bone marrow [53].

Percussive technology was an inherent component in the toolkits of the two earliest and long-lasting (∼1 million years each) lithic techno-complexes, the Oldowan and Acheulian, although their functions may have changed through time [54, 55]. For those early periods, different types of percussive tools (e.g., passive hammers or anvils, hammerstones and pitted stones) have been identified both in and outside Africa [28, 56, 57]. However, in many cases, due to the lack of use-wear analyses on these artefacts, inferences about their specific functions are based on preconceived assumptions about their size and morphology. The problem is exacerbated due to the lack of experimental reference collections for use-wear traces generated by the use of various raw materials. Therefore, the relation between raw material physical properties and artefact function remains unknown.

The two earliest stone tool complexes, the Oldowan and the Acheulian, are currently known to have appeared and proliferated within the East African Rift Valley. The Oldowan (starting at ∼2.6 Ma) represents a fundamental technological breakthrough involving obligatory use of percussive technology, using different types of lithic supports (e.g., cobbles; nodules, pebbles) as percussors for producing simple flakes [58, 59]. The appearance of the Acheulian at ∼1.75 Ma [60–62] arguably marks major biological and behavioural transformations in hominin lifeways, frequently associated with increased cognitive capabilities [54, 63]. Archaeologically, such transformations are recognized through technological and morphological innovations, including the initial appearance of reduction sequence focused on the production of Large Cutting Tools (LCTs) coupled with strict selection criteria for raw materials [64–67]. The extensive temporal and geographic scale of the Oldowan and the Acheulian within Africa and beyond reflects the successful technological and ecological adaptations of their respective makers. The enabling environmental and physical factors and mechanisms for such success are therefore worthy of further investigation.

Here we present results from the first stage of an ongoing study of raw material economy that aims to describe the physical properties and explore the strategies for the selection and use of different raw materials for percussive elements by the Acheulian tool-makers in eastern Africa. This approach focuses on the characterization of raw materials, which we consider fundamental for further functional analyses of the archaeological assemblages. The core case study for this research is the Acheulian site-complex of Melka Wakena, in the assemblages of which we have identified a large number of percussive tools made from various raw materials.

## 2. The case study of Melka Wakena site-complex (Ethiopia): Archaeological background

**Fig 1:**
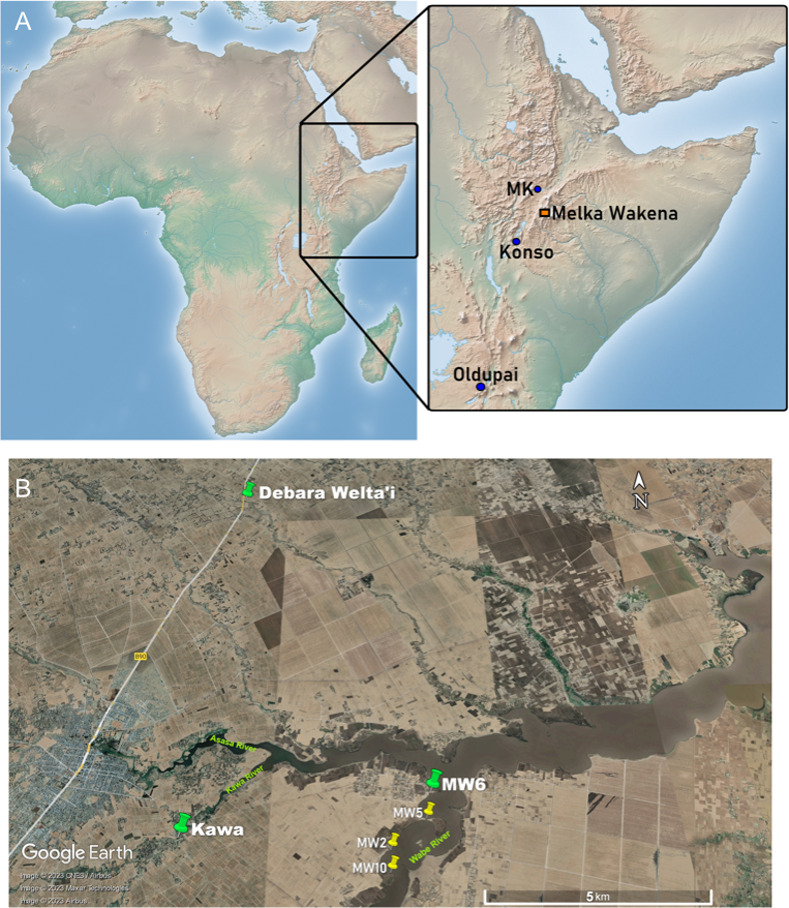
A) Map showing the location of the Melka Wakena site-complex. Inset shows some of the eastern African sites mentioned in the text. Relief map from Natural Earth (public domain): http://www.naturalearthdata.com/. B) Map showing the location of raw material exposures, where the samples for this study were collected (indicated by red dots) and some of the archaeological localities (marked by pink dots). Ignimbrite was sampled from MW6, Glassy ignimbrite from Kawa, and Pumiceous ignimbrite, basalt, and scoria from Debara Welta’i. Terrain map from USGS National Map Viewer (public domain): http://viewer.nationalmap.gov/viewer/.

Melka Wakena is an early Acheulian (∼1.6 to >0.7 Ma) site-complex located in south-central Ethiopian Highlands at an elevation of 2300–2350 m above sea level (Fig 1). It consists of several localities within approximately a 2-km stretch along the western bank of the Wabe River [66, 68, 69]. Preliminary investigations revealed faunal remains, including 15 species of large vertebrates with some animal bones bearing anthropogenic marks (under further investigation).

The lithic assemblages document reduction systems for both small debitage and LCTs made on large flakes, each associated with different raw material procurement strategies, as well as high frequencies of percussive tools. The lithic assemblages were made on various pyroclastic rocks, specifically various grades of ignimbrites, basalt, and scoria. These demonstrate considerable variation in terms of sizes and morphologies (S4 File) and were used selectively for the various lithic morphotypes in all the localities over the time range of 1.6 Ma to 0.7 Ma. [66–69]. However, the relationship between artefact characteristics and the physical properties of the raw materials, and how such properties are linked to the functional use of the artefacts, is still unknown.

Our investigation centers on examining how variability in the physical properties of raw materials may have affected the selection, design, and use of percussive technology during the early Acheulian human occupations at Melka Wakena. To achieve this, we have conducted second-generation experiments using a mechanical experimental setup and standardized samples to evaluate the mechanical performance, including efficiency and durability, of the various raw materials available at Melka Wakena. By controlling for confounding variables such as uneven tool shape or surface morphology, this approach has allowed us to directly measure the performance of the raw materials. This method also enables us to address questions regarding the influence of raw material properties on their selection, manipulation, and use by early hominin populations. While our current research focuses on the case study of raw material variability at Melka Wakena (MW), the results have broader implications for other geographic and chronological contexts where percussive technology has been reported in the archaeological record.

In this study, our investigation focuses on two main aspects: 1) the physical characterization of the different types of rocks present at the archaeological site-complex of MW, and 2) testing the mechanical performance, fracture mechanics and use damage patterns on the different raw materials.

## 3. Material and methods

### 3.1. Raw material sampling

The MW site-complex is situated at 2300–2350 m above mean sea level at the headwater of the Wabe Shebele drainage system (Fig 1), in sediments associated with the early Pleistocene Dino Formation [67, 70]. Located in an area commonly known as the "Gadeb plain", the stratigraphic sequence consists of primary pyroclastic deposits and interbedded fluvial sediments, as well as reworked volcaniclastic sediments. The upper portion of this sequence is exposed by the Wabe River and its tributaries in the Melka Wakena research area. The origin of volcanic rocks on the Gadeb plain is in general associated with the formation and activity of the shield volcanoes on the eastern shoulder of the Main Ethiopian Rift (MER) during the late Pliocene and early Pleistocene [70, 71]. These volcanic centers produced proximal trachytic and basaltic extrusives, as well as pyroclastic (ignimbrite) flows and distal pyroclastic fall deposits (see Resom et al. [2018] for the field, petrologic, major, and trace element geochemical study).

This study includes all the lithic raw materials identified in the archaeological assemblages that are local volcanic rocks, in particular basalt, scoria, and three types of ignimbrites (“regular”, glassy, and pumaceous) (S4 File). These raw materials are not evenly distributed in the landscape within and around the site-complex. Flow deposits of ignimbrite of various grades are extensively exposed along the banks of the Kawa, Asasa and Wabe Rivers (Fig 1), within a 4–5 km radius around the archaeological localities. Outcrops consisting of basalt or scoria, which differ in size and shape, are currently widely distributed to the north of the MW site-complex. Small and coarse-grained pebbles of basalt are generally characterized by ranging from theolitic to alkaline compositions [70]. Scoria is also present in and around the MW localities. These are less abundant relative to the various types of ignimbrites. They are probably sourced from longer distances and transported to the vicinity of MW localities by the network of channels active on the landscape.

The various types of ignimbrites have been identified and distinguished primarily based on their mineralogical composition [70]. Glassy ignimbrite is differentiated by the presence of a glassy matrix, minor quartz crystals, and finer grains [70, 71]. The rock presents a colour gradient from light to dark green. Regular ignimbrite is found as strongly fractured, weathered, and sub-rounded to sub-angular blocks, although it shows similar mineral constituents and modal composition as glassy ignimbrite. While it yields sharp edges upon breaking, it exhibits characteristics compatible with a lesser degree of welding compared to the glassy ignimbrite. The pumaceous ignimbrite contains slightly vesiculated pumice clasts. This mostly gray-coloured rock shows a moderate degree of welding (i.e., it is lighter than the other two ignimbrite types) and easily crumbles upon breakage [70, 71].

For this study, samples of these raw materials were identified in the field by the naked eye and under a magnifying glass and collected from various locations within and around the MW site-complex in a systematic survey conducted in 2021 and during the 2023 field season covering distances of up to 10 km from the archaeological localities.

### 3.2. Sample preparation

From the raw materials collected in the field, we have prepared standardized samples in terms of morphology and dimensions. A total of 5 cubes measuring 2.5x2.5x2.5cm (1 cube for each raw material) were sliced out from the interior of each rock using a diamond rock saw, seeking to standardize the surface conditions to acquire the hardness data and to exclude the effect of the natural morphology of raw materials during the experiments (details in section 3.3). We have engraved small dots on each cube’s face in order to use them as reference points to support the alignments of the scans and relocate areas of interest.

### 3.3. Raw material physical characterization (hardness and internal structure)

The selected raw materials are described here in terms of density, Leeb rebound hardness and internal structure. The density of the different rocks was measured on the standard saw-cut experimental samples. To measure density, the mass per unit volume (density = Mass / Volume) was calculated. Each sample was weighed, and the volume was obtained from the 3D models. Raw material hardness was measured using the non-destructive Leeb Rebound hardness tester equipped with a Leeb C probe (Proceq Equotip 550). This portable test employs a technique that calculates the energy loss of an impact body from impacting a second body. In this test, the impact body rebounds faster from harder samples than from softer ones, expressed by the Leeb rebound hardness unit HLC.

To evaluate the internal and external variability and validity of all different samples, we took a total of ten measurements, at different positions on each sample cube. As part of the Proceq equipment requirements (sample size and mass limits), due to the small size of the experimental samples, all hardness measurements were acquired with the sample positioned on top of a flat, heavy and stable block (in this case a granite slab using coupling paste).

The five used cubes were scanned with a Phoenix V|tome|x L CT-scanner (Baker Hughes / Waygate Technologies) at the Leibniz-Zentrum für Archäologie (LEIZA), to assess the characteristics of the internal structure of the raw materials. The microfocus tube was used at 110 kV and 250–270 μA, yielding 27.5 W. The focal spot size is roughly equal to the power, i.e. 27.5 μm. Voxel size was geometrically set to 22 μm. Such values for power and voxel size are in an optimal relationship with each other to ensure adequate levels of geometric unsharpness. Acquisitions and reconstructions were performed in datos|x. All acquisition and reconstruction settings can be retrieved from the PCA and PCR files, respectively, available for each scan on Zenodo (10.5281/zenodo.10631628). These files can be opened with any text editor.

### 3.4. Experimental design

The experiments aim to test the surface alterations of the different raw materials present in the archaeological assemblages at MW when submitted to the same motion and compare the damage patterns. To measure the mechanical properties of the different raw materials, we designed a second-generation, controlled laboratory experiment. In our experimental study, we investigate the null hypothesis (H_0_) *that differences in raw material physical properties do not affect the degree of damage (and durability) caused by the tool’s use*. Specifically, we tested the causal relationship between the raw material (independent variable) and the degree of damage on the sample (dependent variable/outcome).

**Fig 2:**
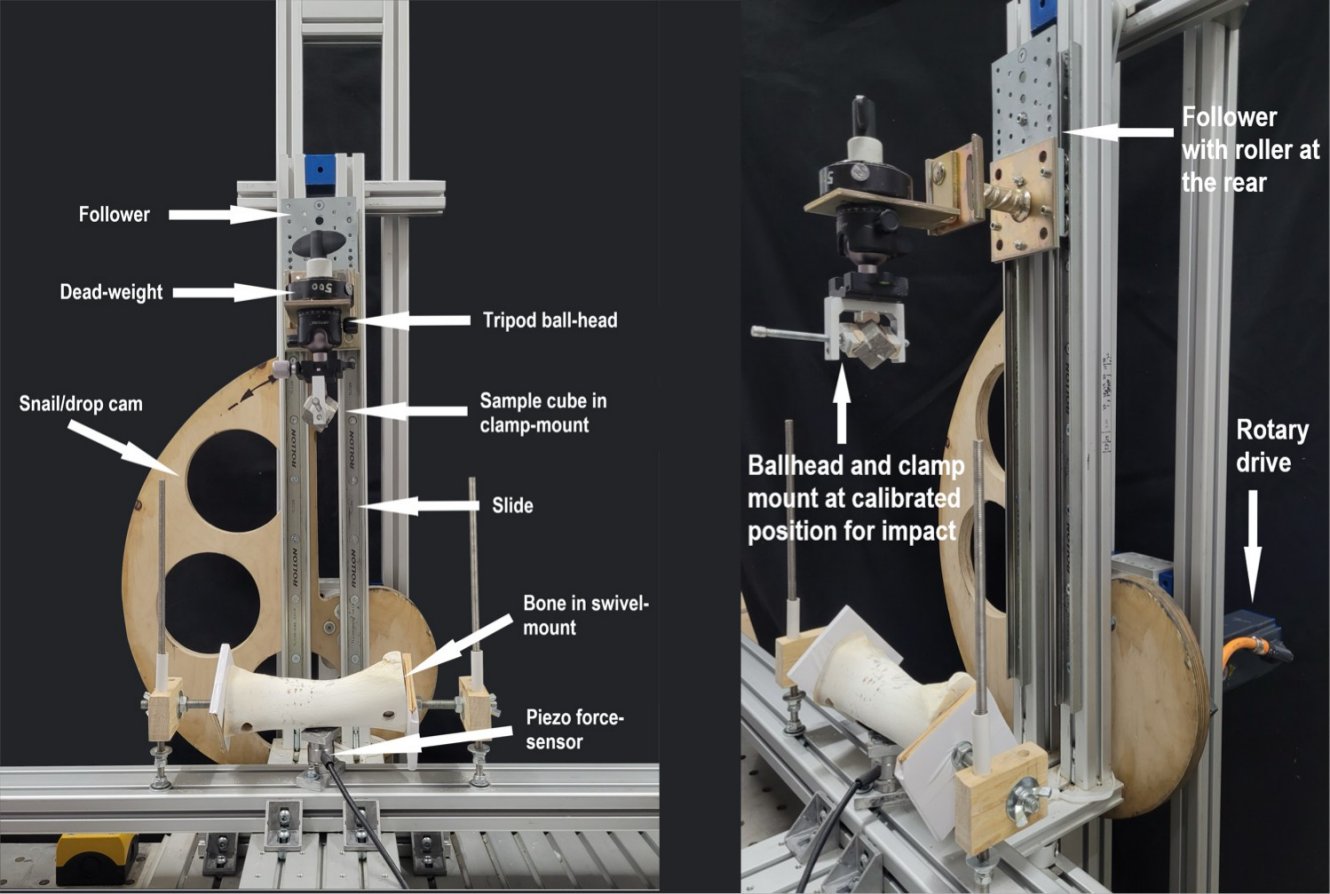
Experimental mechanical setup.

**Fig 3:**
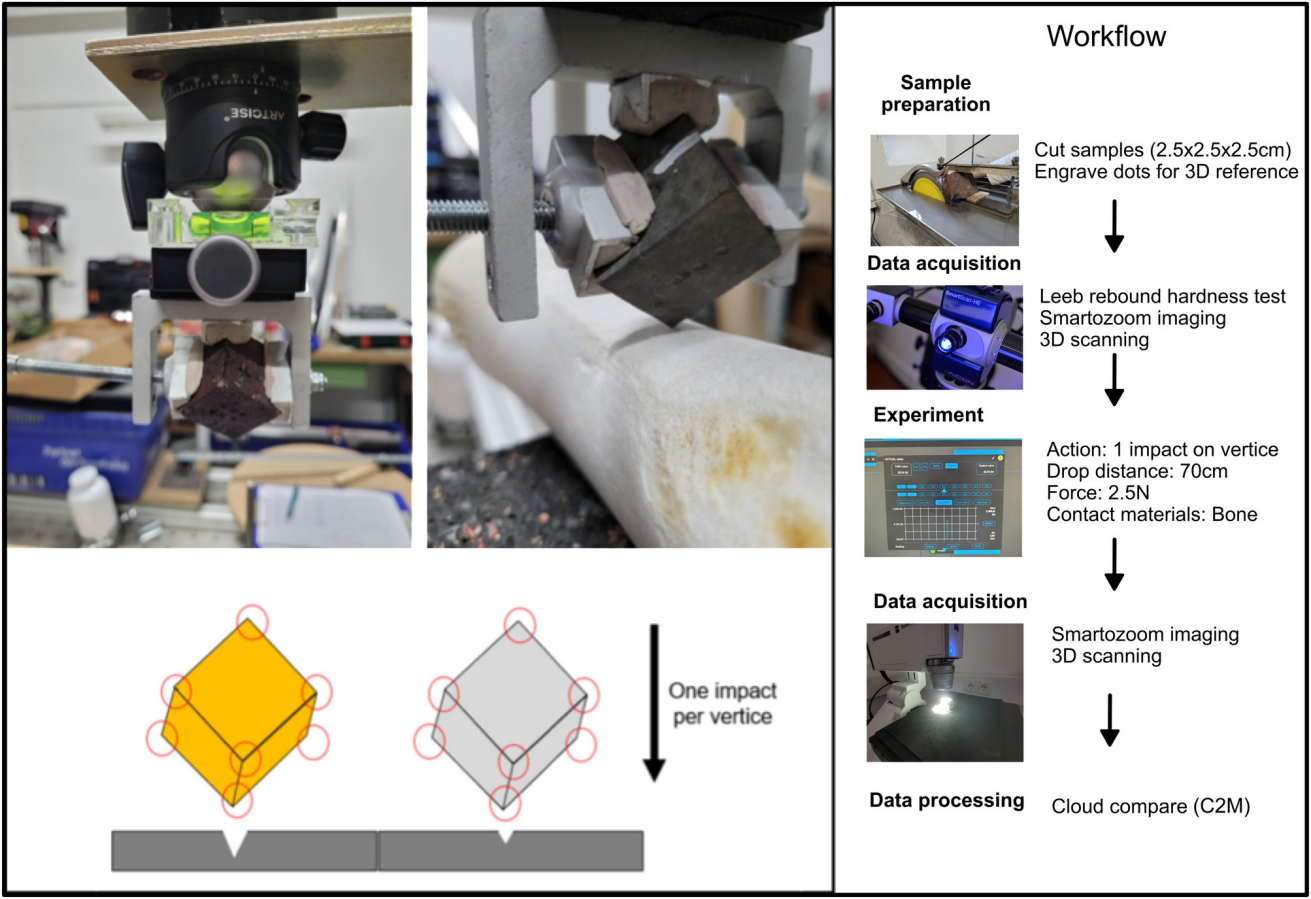
Simplified workflow of the experiment and data collection.

Here we focused on comparing the level of alterations on the lithic raw materials (the ‘percussors’ in the experiment) when submitted to the same stress. In this case, we used an impact on a cow’s long bone. On each cube, four vertices (samples) were used for a single impact, each on a clean bone surface. The impact force was standardized by keeping the same weight (2.5 kg including sample holder) and dropping distance (28 cm). The cube was clamped into a vice on a sample holder specially designed for this experiment, using epoxy resin putty to adjust the cube to a standard and stable position (Figs 2 and 3).

The experiment, dedicated to mechanically testing the different raw materials, was developed using a mechanical device (SMARTTESTER^®^, manufactured by Inotec AP GmbH, with adaptations made by Walter Gneisinger). This customized setup allowed us to define and record multiple parameters including force and the number of impacts [39, 72]. The setup allows a systematic lift and drop of a sample from a predeterminate vertical distance onto a passive material, using an automated rotary drive and a snail/drop cam with a follower controlled by a central computer (see Fig 2). The passive material sits on top of a force sensor connected to the same central computer. The experiment was done by applying standardized impact, while the previously defined variables–impact force (measured peak force of 2.5kN), number of impacts (1 per vertex), and the position of the sample–were kept constant throughout the entire duration of the experiment. Samples were used as a contact point for the impact on the bone.

### 3.5. 2D imaging and 3D analysis (Fig 4)

**Fig 4:**
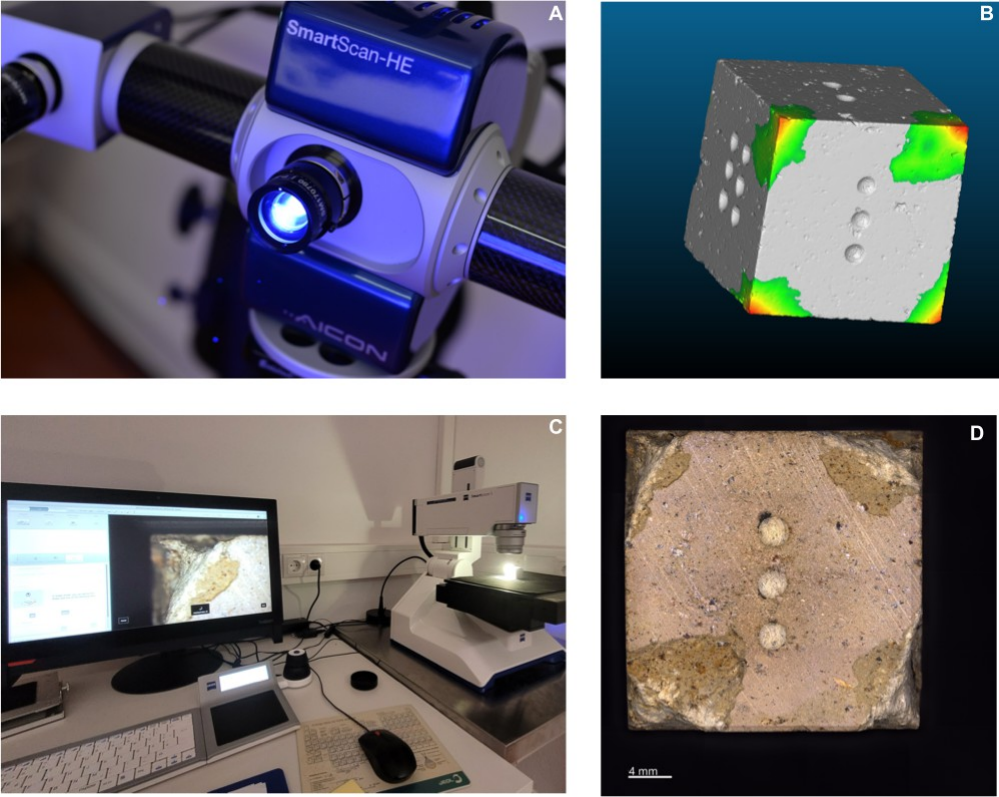
A) Aicon smartSCAN-HE R8 B) Visualization of impacts on *cloudcompare* software C) macroscopic digital imaging with Zeiss SmartZoom5 D) Example of sample imaging after experiments.

In our study, we combine qualitative and quantitative approaches to document our samples more holistically, the documentation and analysis of the experimental samples were carried out using both 2D and 3D imaging techniques. To measure and evaluate the durability of all raw materials, our analysis focuses on two main aspects: 1) qualitative surface damage* ‐ macroscopic observation*, 2) analysis of quantitative volume loss of the raw material samples. All experimental cubes were scanned before and after the experimental cycle using a high-resolution 3D light structure Scanner (Aicon smartSCAN-HE R8) (see S1 File for details on the acquisition settings). All samples were extracted and analyzed individually. The 3D models were exported as .*ply* files and imported into the *CloudCompare software V2*.*12*.*3*. Comparison of the 3D scans (before and after use) of each sample allows us to quantify the level of damage (volume loss) in each raw material cube. This was done by measuring the distances between the 3D point clouds. For this approach, we used the Cloud-to-Mesh (C2M) distance tool, which computes the distances between 3D models (see S2 File for the workflow) [36, 44]. For each experimental sample we refer to the 3D meshes before use (no damage) and after use (with damage) as “Reference model” and “Compared model”, respectively. We used for all samples a step value of 256, which means that all models are equally segmented into 256 parts. Data were exported and combined in a single dataset. The total amount of distance was calculated for each pair of samples (before and after use). The total amount was then organized by sample and raw material, and projected in a boxplot.

The distance between C2M is computed as the absolute Hausdorff distance, hereafter aHd (also called Pompeiu–Hausdorff distance) [36, 73]. To reduce the noise caused by the natural error margin of the alignments we have applied a threshold of 0.5 mm in the classification of the cloud distances. The computation calculates the distance between 3D models (before and after use) for each segmented part. After comparison, the generated data from each computation was exported and combined in a single .csv file and imported into an R script for the statistical analyses (see S3 File).

After computation, the result of the comparison displays the distance on a colour scale. The colder colours depict low distance values (i.e., target specimen is a close match to the reference specimen) and progressively warmer colours depict increased distances between the two states on the specimen. The distribution of the absolute distances is visualized through a histogram. The computation calculates the distance between each part of the two meshes.

The observation, analysis and documentation of the samples’ surface before and after use was carried out using a digital microscope Zeiss SmartZoom5 equipped with a 1.6x/0.1 objective (see S1 File for details on the acquisition settings). Images were collected at a total, on-screen magnification of 34x.

In addition, CT-scans were used to assess a detailed inspection on (i) the effect of the impact experimentation on the samples’ internal structure and (ii) the effect of the rocks’ internal structure, e.g., pores, oriented minerals etc., on the impact. Regrettably, the study started without access to CT-scan technology, resulting in a lack of pre-experiment CT-scan data. Future investigations will benefit from comparing CT-scan data collected before and after experimentation, facilitating a more comprehensive and detailed analysis of the alterations within the internal structure of each raw material. When needed, digital images (including overviews, areas of interest, and particular features) were edited (e.g., cropping and resizing) after the acquisition, using GIMP (free open-source image editor, available at https://www.gimp.org/, v.2.10.18). The combination of different images in a single figure was processed using the software Inkscape (free and open-source vector graphics editor, available at https://inkscape.org/, v.0.92.4).

### 3.6. Data processing and analysis

Descriptive statistics were calculated for the hardness values and densities of all raw materials and 3D measurements of the degree of volume loss (using aHd measurements), for comparison within and between samples. Because in this laboratory-controlled experiment, the sample size is small (4 vertices per raw material) and therefore, its distribution cannot be accurately assessed, we safely used a non-parametric Kruskal–Wallis H test (bootstrapped over 1000 iterations), and the resulting 95% confidence interval of the p-value, to assess differences in volume loss (aHd) between raw material samples. We followed the method recently published by Lin et al. (2023), here adjusted for more than 2 predictor variable groups. Kruskal-Wallis rank sum test checks if the null hypothesis that the observations on volume loss are the same for each raw material. The alternative hypothesis is that they differ for at least one raw material. Additionally, Kendall’s Tau correlation test was used to check the strength and direction of a relationship between two variables. The value of a correlation coefficient ranges from -1 to 1. "-1" indicates a perfect negative relationship; "0" indicates no relationship between variables; "1" indicates a perfect positive relationship between variables. All data analysis and plotting were processed with the R open-source software (see S3 File for detailed info on used packages and software versions, scripts, raw and processed data.

A research compendium, using the rrtools package by Marwick et al (2018), including detailed info on used packages and software versions, raw and processed data is available here: https://doi.org/10.5281/zenodo.10631628, under the MIT license, data under CC-0, and figures under CC-BY (see further details in Marwick, 2017).

## 4. Results

### 4.1. Raw Material description and physical characterization

The five selected and used raw materials in our study show distinct physical properties, concerning their density, Leeb rebound hardness (HLC), and internal structure.

**Fig 5:**
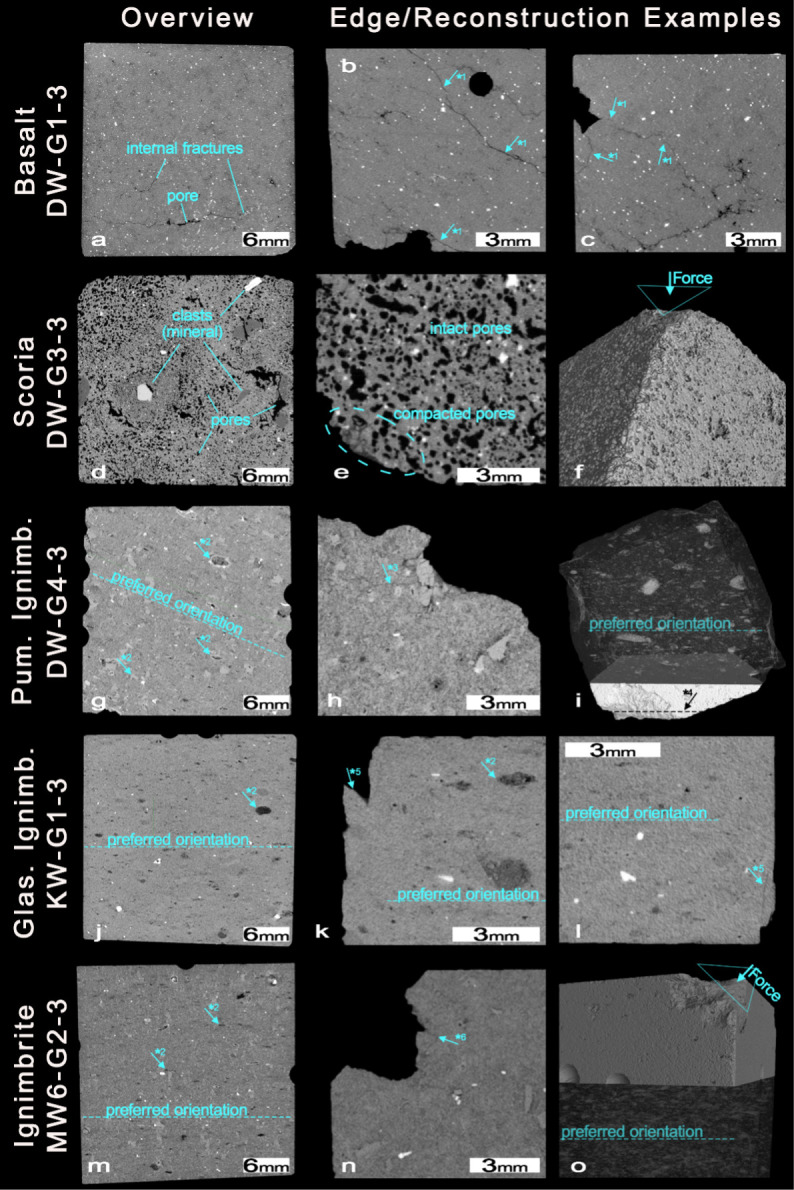
CT data of the used cubes with overviews as centred cross section in the 1^st^ column and close-up views on the cube’s impact corners in the 2^nd^ and 3^rd^ columns. a. The basalt cube with pores connected to the internal fracture network. b-c. Close-up view of impact edges with breaking along or parallel to internal fractures (*1). d. The scoria cube characterized by a high abundance of pores and clasts of various lithology. e. Compacted pores off an impact corner. f. Reconstruction of impact corner showing its parallel surface to the impact surface. g. The pumaceous ignimbrite cube with a medium-strong preferred orientation formed by clasts and pores (*2). Partly, the pores show rims of remaining mineral h. Close-up view on impact corner with large material loss and induced impact fractures (*3). i. Reconstruction of the pumaceous ignimbrite cube with the upper part showing the preferred orientation of high CT-number clasts and the lower part showing the breaking edge of an impact corner which is parallel to the preferred orientation (*4). j. The glassy ignimbrite with a weak preferred orientation by pores and clasts (*2). k-l. Close-up view on impact corners with small scale chipping off at breaking edges in high angle to the preferred orientation (*5). m. The “regular” ignimbrite with medium-strong preferred orientation of pores and clasts. n. Close-up view on impact corner with large material loss and induced impact fractures (*6). o. Reconstruction of an impact edge with the impact surface not affected by the preferred orientation inside the cube shown in the lower half of the cube.

By using computed tomography (CT) the internal structure as well as its effect on the impact were investigated. The CT Fig 5 shows in the 1^st^ column overviews as centered cross sections of the investigated cubes. Here, primary distinguishing features between the lithologies are recognizable; these are (i) the presence of pores and clasts, (ii) the presence and strength of a rock fabric which is formed by a preferred orientation of the pores or minerals and (iii) the presence of internal fractures (Table 1). The views on the cube’s corners in the 2^nd^ and 3^rd^ columns illustrate the effect of the impact on the material.

**Table 1:** Internal material features and the presence of impact fractures based on CT data.

SAMPLE		MATERIAL FEATURES				
Rock type	Sample name	Preferred orientation	Visible clasts	Pores	Internal fractures	Impact fractures
Basalt	DW-G1-S3	-	-	few (connected to fractures)	yes	minor
Scoria	DW-G3-S3	weak *^1^	yes	frequent	-	-
Pumaceous Ignimbrite	DW-G4-S3	medium*^2^	yes	few (dissolved minerals)	-	yes
Glassy ignimbrite	Kw-G1-S3	weak^*2^	yes	few (dissolved minerals)	-	yes
Ignimbrite	MW6-G2-S3	medium^*2^	yes	few (dissolved minerals)	-	yes

Preferred orientations were formed primarily by pores (*^1^) or minerals (*^2^).

The basalt is characterized by low porosity and a fine matrix with little to no visible clasts. The existing pores are connected to internal fractures which mostly follow a preferred orientation (Fig 5A). Breakouts at the impact corners follow these existing internal fractures (Fig 5B and 5C *1). In places, the chips breaking off are not located at the impact corner but along internal fractures at a distance from that corner (Fig 5C). The scoria is characterized by the highest porosity and clasts of different lithologies (Fig 5D). The shape of the pores forms a weak preferred orientation, not visible in the section shown. At the corners of the impact the pores collapse, and the debris is compacted (Fig 5E). The resulting flat surface corresponds to the “intruding” flat surface of the long bone (Fig 5F).

All three ignimbrite types have a preferred orientation of pores and clasts (Fig 5G, 5J and 5M). The pumaceous ignimbrite shows the strongest preferred orientation. Relicts of mineral material in some pores suggest that the pores result from mineral dissolution. Small fractures originate from the corners of the impact (Fig 5H*3). For some impact corners, the breaking edge follows the preferred orientation (Fig 5I*4). For the glassy ignimbrite, this linkage is not present. Sharp breaking edges at the impact corners are at a high angle to the preferred orientation (Fig 5K and 5L). At these small-scales, chipping-off along newly formed impact fractures is visible (Fig 5L). The regular ignimbrite exhibits a stronger foliation than the glassy ignimbrite (Fig 5M). Nevertheless, the breakout of material at impact corners does not seem to be dependent on this orientation (Fig 5O). In fact, extensive crushing of the material occurs along newly formed impacted fractures reaching into the material (Fig 5N*6). The shape of the impact corners is partly flat resembling the intruding long bone surface.

**Fig 6:**
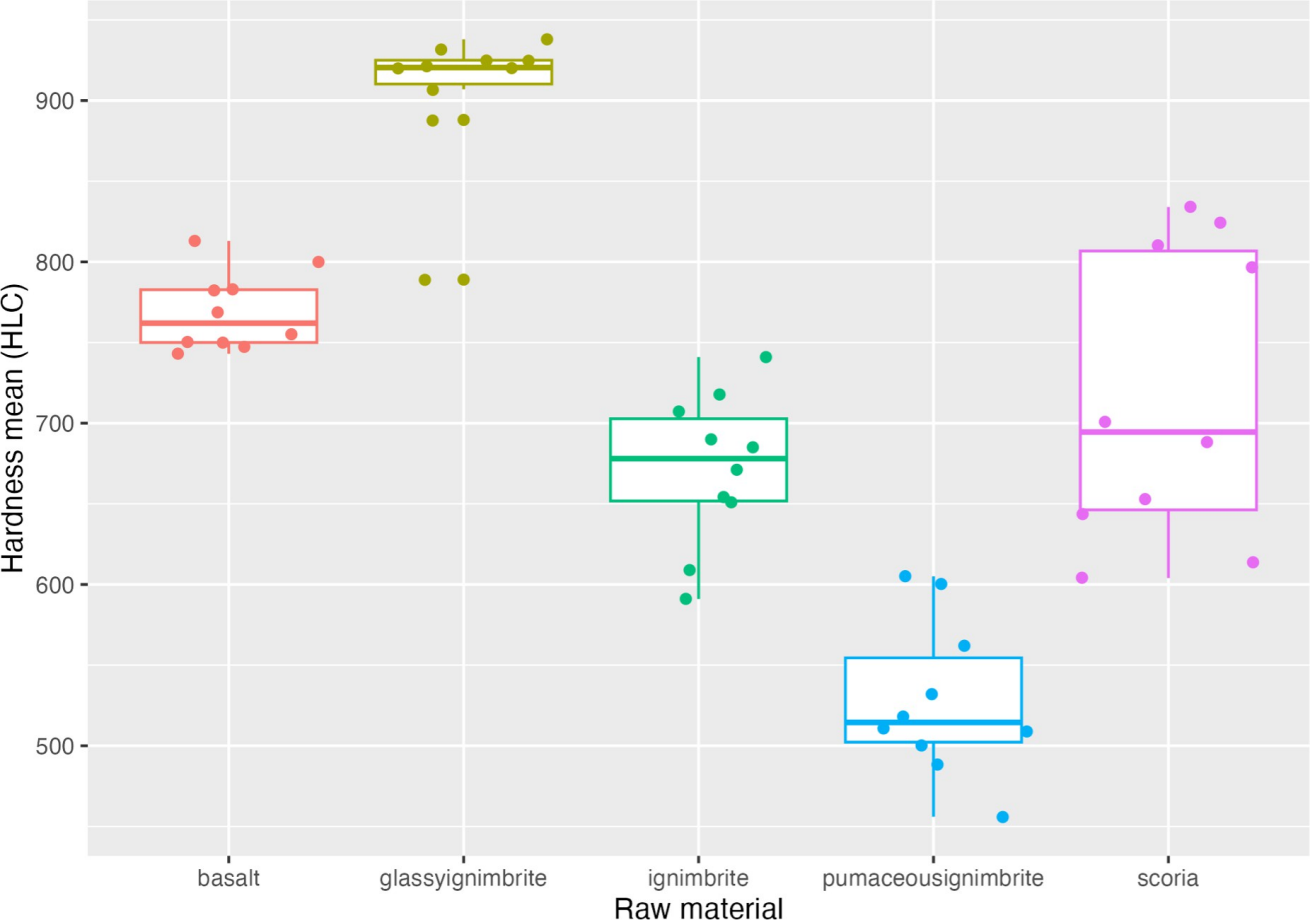
Leeb rebound hardness values boxplots.

Concerning the Leeb rebound hardness measurements (Table 2), ignimbrite shows a large dispersal, with values ranging between 591 and 741 HLC. The glassy ignimbrite is the most homogenous (values between 789 and 938 HLC), as indicated by the lowest standard deviation (43.5 HLC) (Table 2). The values for pumaceous ignimbrite (between 456 and 605 HLC) are clearly the lowest of all raw materials both in hardness values and in density, with a value of 2,32. Basalt presents hardness values in the range between 743 and 813 HLC. This is the material with higher density, with a value of 3.53 (Table 2) Finally, the scoria has hardness values in the range between 604 and 834 HLC, and therefore is the most heterogeneous of the raw materials (see Fig 6).

**Table 2:** Hardness and density data by raw material.

Cube ID	Raw material	Hardness (counts)	Hardness (min)	Hardness (max)	Hardness (mean)	Hardness (median)	Hardness (sd)	Mass (gr)	Volume (cm3)	Density
**DW-G1-S3**	Basalt	10	813	743	769.2	24.35752222392271	762	55.22	15.63	3.53
**DW-G3-S3**	Scoria	10	834	604	716.9	90.72969622884108	694.5	46.62	15.63	2.98
**DW-G4-S3**	Pumaceous ignimbrite	10	605	456	528.1	47.84570107241727	514.5	36.28	15.63	2.32
**KW-G1-S3**	Glassy ignimbrite	10	938	789	906.5	43.52585438564073	920.5	46.83	15.63	3
**MW6-G2-S3**	Ignimbrite	10	741	591	671.7	47.01075527058793	678	39.55	15.63	2.53

### 4.2. Surface alterations

#### 4.2.1. Qualitative assessment

Macroscopic observations, analyses, and documentation of surface alterations (e.g., macro damage) on each raw material before and after use were carried out using a digital microscope. Our qualitative assessment of the macro damage allows us to identify different degrees of damage between raw materials. Pumaceous ignimbrite is highly altered, showing a high level of crushing and flake scars that extend through the surface outside the impact area. This high degree of damage correlates with its low hardness values.

**Fig 7:**
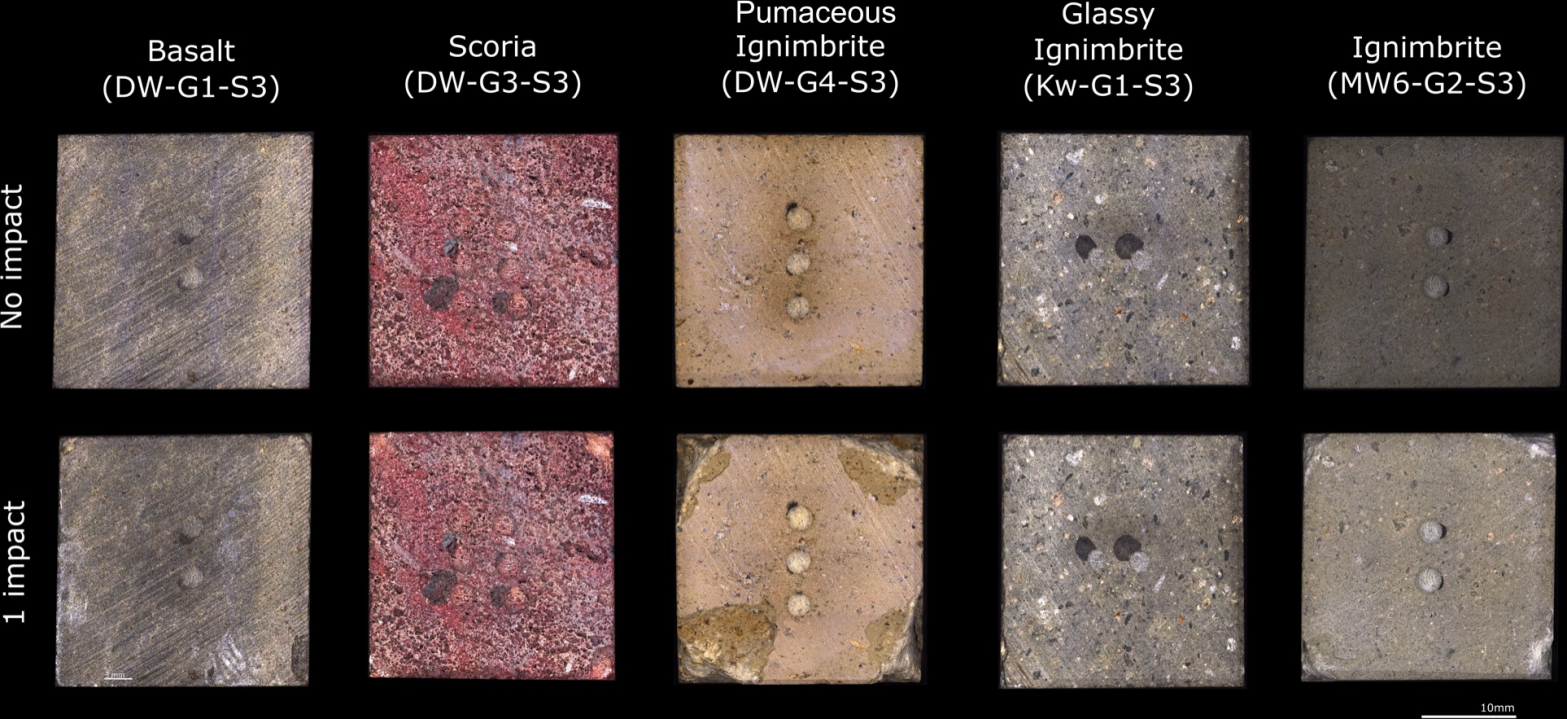
Macroscopic digital image with ZEISS SmartZoom, showing a comparison of before and after the experiment.

While still visible at this scale of observation, the damage on the basalt, ignimbrite and scoria is much less developed in comparison with the pumaceous ignimbrite (see Fig 7). In the case of basalt, some minor micro-flaking in one of the samples is still visible, yet only minimal crushing is present solely at the impact area.

Our observations suggest that the glassy ignimbrite shows a very low degree of damage, almost undetectable at this macroscopic scale of observation and analysis. This result inversely correlates with the higher value of hardness when compared with all the other raw materials (> 900 HLC).

#### 4.2.2. Quantitative 3D volume loss analysis of the raw material samples

**Fig 8:**
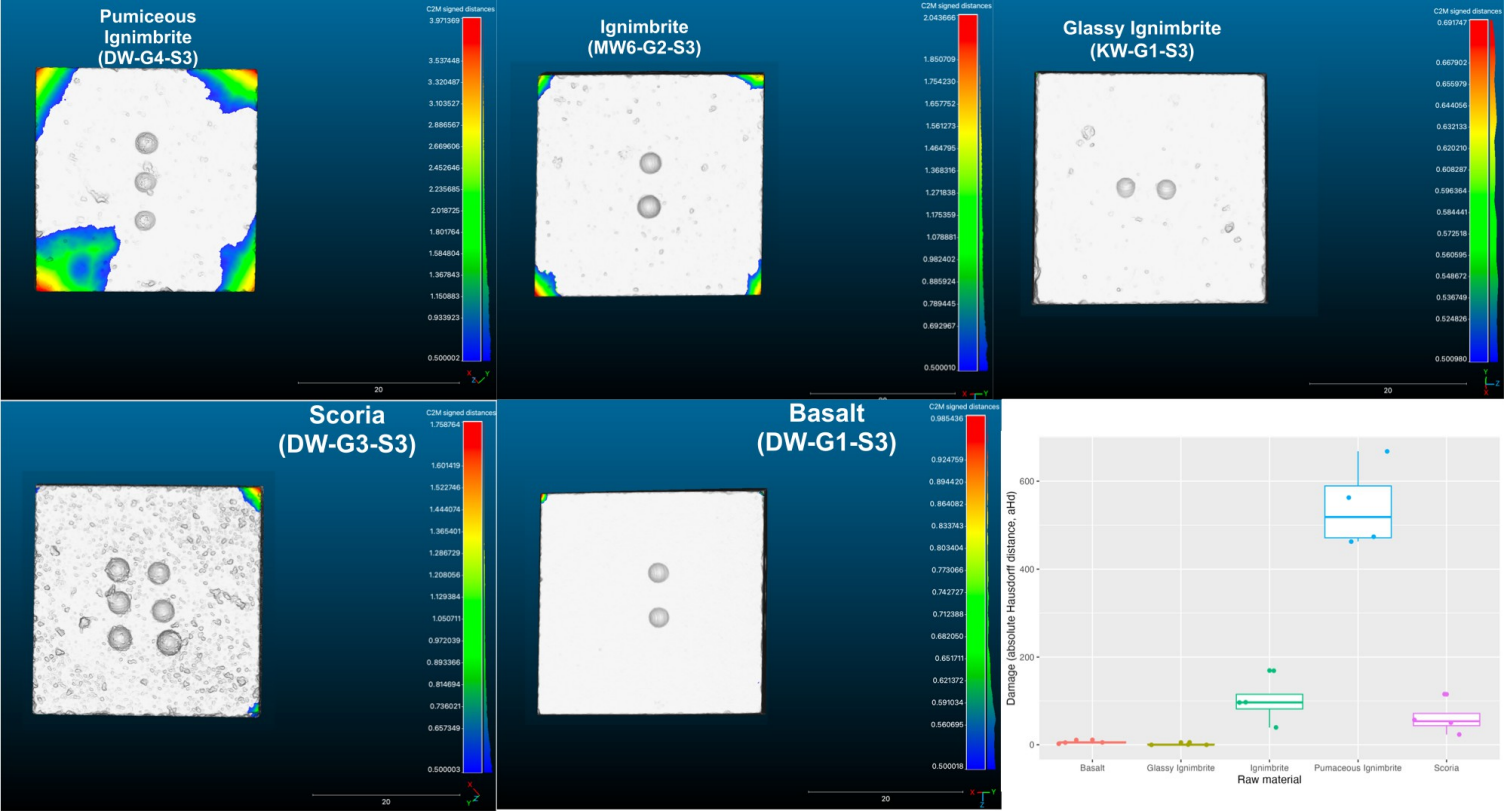
Visualization of the volume loss quantification for each of the raw material (using the C2M tool in *CloudCompare software V2*.*12*.*3) and respective boxplots of the total volume loss (damage) between before and after the experiment*.

To complement the qualitative description of the surface damage, the quantitative assessment of the degree of damage on each sample after use was measured (section 3.4). Table 3 summarizes the volume loss of the raw material samples. The collected data show a clear difference in the amount of damage between raw materials (Fig 8 and Table 3). The pumaceous ignimbrite reveals by far the highest degree of damage followed by the ignimbrite and the scoria. The latter two raw materials show similar average values. On the other hand, the basalt and the glassy ignimbrite are the most resistant to surface alterations and show the lowest degree of damage. According to the bootstrapped Kruskal-Wallis rank sum test, the null hypothesis that the degree of volume loss is the same in each raw material can be rejected (p-value 95% confidence interval: 0,0010–0,0021), which indicates that at least on one sample (raw material) the degree of damage is significantly different from the others. The Wilcox test for pairwise comparisons between groups shows that only glassy ignimbrite and basalt are not significantly different (p-value > 0.05), and that pumaceous ignimbrite and scoria are significantly different from each other, and from all other raw materials (p-value < 0.05).

**Table 3:** Volume loss data by raw material and sample.

Cube ID	Sample	Raw material	Cycle	Volume loss (counts)	Volume loss (max)	Volume loss (min	Volume loss (mean)	Volume loss (sd)	Volume loss (median)
DW-G1-S3	DW-G1-S3-V1	Basalt	0–1	256	5	0	1.140625	1.2817535476302553	1
DW-G1-S3	DW-G1-S3-V2	Basalt	0–1	256	11	0	3.7890625	2.3685930091801874	3
DW-G1-S3	DW-G1-S3-V3	Basalt	0–1	256	2	0	0.06640625	0.2791520335643953	0
DW-G1-S3	DW-G1-S3-V4	Basalt	0–1	256	5	0	1.296875	1.16052303962058	1
								26	
DW-G3-S3	DW-G3-S3-V1	Scoria	0–1	256	23	0	6.0078125	3.713243059591958	5
DW-G3-S3	DW-G3-S3-V2	Scoria	0–1	256	115	2	39.76171875	27.69600132841081	34
DW-G3-S3	DW-G3-S3-V3	Scoria	0–1	256	57	0	9.4296875	15.682228529984718	1
DW-G3-S3	DW-G3-S3-V4	Scoria	0–1	256	50	0	15.3828125	12.014123122881864	12
DW-G4-S3	DW-G4-S3-V1	Pumaceous ignimbrite	0–1	256	563	9	174.2890625	158.78838562474814	93.5
DW-G4-S3	DW-G4-S3-V2	Pumaceous ignimbrite	0–1	256	474	7	154.4296875	120.11061461272713	106.5
DW-G4-S3	DW-G4-S3-V3	Pumaceous ignimbrite	0–1	256	463	10	160.54296875	121.56004773472907	112
DW-G4-S3	DW-G4-S3-V4	Pumaceous ignimbrite	0–1	256	668	7	178.484375	161.6882977746222	107.5
KW-G1-S3	KW-G1-S3-V1	Glassy ignimbrite	0–1	256	5	0	1.22265625	1.0219331810204562	1
KW-G1-S3	KW-G1-S3-V2	Glassy ignimbrite	0–1	256	0	0	0	0	0
KW-G1-S3	KW-G1-S3-V3	Glassy ignimbrite	0–1	256	0	0	0	0	0
KW-G1-S3	KW-G1-S3-V4	Glassy ignimbrite	0–1	256	0	0	0	0	0
MW6-G2-S3	MW6-G2-S3-V1	Ignimbrite	0–1	256	97	2	25.2890625	19.11580901797684	19
MW6-G2-S3	MW6-G2-S3-V2	Ignimbrite	0–1	256	39	0	19.33984375	8.031172989254642	20
MW6-G2-S3	MW6-G2-S3-V3	Ignimbrite	0–1	256	169	6	70.59765625	40.80760975716585	61.5
MW6-G2-S3	MW6-G2-S3-V4	Ignimbrite	0–1	256	96	2	26.03125	17.728425164789158	23

**Fig 9:**
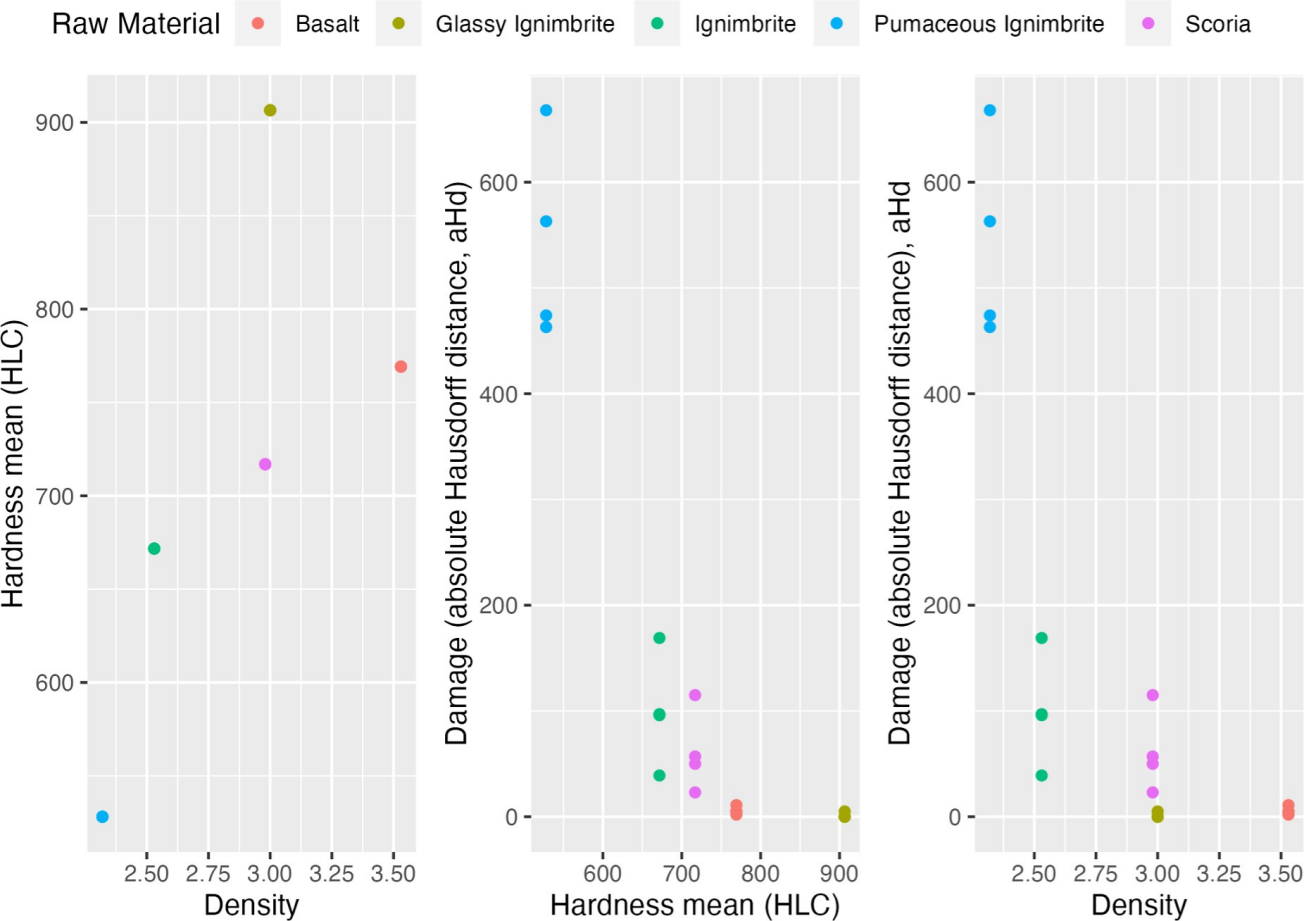
Scatterplots showing the relationship between hardness and density, hardness and volume loss, and density and volume loss for all raw materials.

On the other hand, both Hardness (tau = -0.85) and density (tau = -0.71) seem to indicate a negative relationship with the degree of volume loss (Fig 9). These results suggest indeed that high hardness and density values statistically relate to low values of volume loss.

## 5. Discussion

Decision-making processes of early hominins can be inferred from the archaeological record of stone tools. In this particular context, the decisions may be related to major factors, e.g., the availability and procurement strategies of resources, but also to their physical properties that are relevant to the tasks at hand. Differences in the physical properties of rocks are often described and organized by researchers in distinct categories of “raw material quality”; however, defining and quantifying this “quality” is not straightforward. Some researchers emphasize properties that enhance raw material “knappability”–namely, predictability and ease of fracture while being flaked as the main selection criteria. If, however, the raw materials were selected with a view to anticipated tool use, other aspects such as durability or performance are likely to have been prominent in the assessment of “quality” of a given raw material.

While recent research has explored the relation between “raw material quality” and knappability via experimental replications, the impact of variable raw material properties on tool usage still requires systematic investigation with comparable methodologies. In the case of percussive tools, properties of the stone that determines knappability may be less relevant and selection criteria may have been focused on physical properties (e.g., density, hardness) directly related to their effectiveness in transferring force onto various materials.

The combination of analytical tools and methods used in this study is possibly the first of its kind and our experimental results show that when performed on the same contact materials, with the same force and the same number of movements, percussive action can lead to a very different surface alteration depending on the physical properties of the raw materials. A high level of surface alteration is not necessarily linked to a high intensity of use or a high level of force during use. Interpretations of alterations on archaeological stone tools thus require a detailed understanding of raw material properties prior to formulating explanatory scenarios of stone tool functionality. The differences in surface alterations also highlight the importance of increasing the reference collection for use-wear identification in order to include more raw material variability and explore the full potential of this approach for archaeological interpretations.

The techno-typological study of lithic artefacts from the early Acheulean site-complex of Melka Wakena has shown clear correlations between specific raw materials and specific tool types [66, 67, S4 File]. Most notably, although ignimbrite was locally and widely available along the banks of the paleo-Wabe, glassy ignimbrite, brought from a longer distance in the form of large flake blanks, was intentionally selected for the manufacture of LCTs, whereas percussive tools (i.e., anvils and hammerstones)) were made often on basalt [66, 67, S4 File].

Our experimental results show that, while two types of rock (ignimbrite and glassy ignimbrite) appear similar to the naked eye, they behave considerably differently. Importantly, the three different types of ignimbrites present rather distinct properties, both in terms of Leeb hardness and in terms of surface alterations. The glassy ignimbrite is significantly harder (mean hardness value of 907 HLC) compared with the other raw materials (see Fig 6). It is also the most homogenous raw material (as indicated by the lower values of the *SD* of the hardness measures) and the most resistant (as shown by the low degree of surface alterations compared to the other raw materials). The surface alterations data for the glassy ignimbrite are close to those of the basalt. At the other extreme, the pumaceous ignimbrite, being the softer (456 HLC) raw material, developed the most extensive damage during the experiment. In our sample, scoria is the raw material with the highest levels of heterogeneity in hardness values. These results seem to indicate that glassy ignimbrite and basalt are more suitable for tasks that involve percussive contact with harder materials. Further work should also look at how raw material variability, and respective physical properties, correlate with longer periods of tool use, exploring aspects of tool efficiency and durability.

The data discussed above suggest that under the conditions of our test it is possible in most cases to detect a correlation between measured Leeb hardness and the resistance of the raw materials to surface alterations, where a low value of hardness is reflected in higher damage (i.e., volume loss) after impact. This is well expressed in the extreme cases of pumaceous ignimbrite (softer material / higher damage) and of the glassy ignimbrite (harder material / lowest damage). Interestingly, our data indicate that material hardness is not always a clear predictor of volume loss. For example, while glassy ignimbrite and basalt clearly differ in their hardness values, that difference is not proportionally reflected in the damage produced after use. This non-linear relationship seems to indicate that other physical properties (e.g., toughness, elasticity, brittleness) might proportionally affect the degree of damage, and, therefore, needs to be further investigated in future work.

The CT analysis enables us to correlate the differences in hardness and surface alteration with internal material properties and internal structure. The basalt, with its small number of pores and the resulting resistivity to material compaction, in combination with the absence of a strong preferred orientation of minerals, is particularly suitable for percussive tools which require hardness and no preferred structural orientation. However, the existence of internal fracture networks in parts of the basalt, which lead to oriented fracturing during the impact, makes a precise selection of the raw material necessary. The high number of pores in the scoria causes strong alteration associated with the compaction of the pores during impact. The non-homogeneous distribution of larger pores and clasts (e.g., minerals) provides an explanation for the heterogeneity in hardness measured in the experiment.

With a preferred orientation formed primarily by cavities of dissolved minerals, the presence of visible clasts including strong absorbing phases and the formation of impact fractures at the impact corners, all three types of ignimbrite share most material features detected by CT. However, their impact alteration and hardness measurements vary strongly between these raw material types. As Resom and colleagues (2017; Resom et al. 2018) have shown, the main microscopic difference between the ignimbrite types lies in its mineralogical composition, and in particular its matrix. Here, the presence of glass, which welds the grains and clasts together, is most likely decisive for high hardness and low impact alterations of the glassy ignimbrite type. On the other hand, the regular ignimbrite is primarily composed of volcanoclastic components like the pumaceous ignimbrite but lacks this bonding and therefore shows lower hardness and high impact alteration. Interestingly, the preferred orientation of pores and minerals seems to play a bigger role for the impact fracturing of the latter ignimbrites.

The combination of analytical tools used in this study led to a detailed quantification of raw material characteristics, exploring how they impacted the production and design of the lithic artefacts. For example, pumaceous ignimbrite exhibits a significantly greater extent of damage in comparison to other raw materials within the assemblages (see Fig 6). Its lower hardness, density, and increased brittleness are visibly apparent and would lead to the prediction that this raw material would not be selected for percussive purposes. Indeed, pumaceous ignimbrite has not been identified by naked-eye examination of the percussive material in the various assemblages of Melka Wakena (S4 File). In the current study, these properties are demonstrated objectively and measured quantitatively.

Other results of our experimental study are less predictable/intuitive and raise new questions about hominin decision-making. We show that glassy ignimbrite is the hardest, most homogenous and most durable raw material in the Melka Wakena lithic assemblages, followed closely by basalt. Yet despite the similarity in mechanical characteristics (see section 4.2.2.), basalt is often a dominant raw material used in percussive activities throughout the Melka Wakena occupation sequence. This pattern can be explained as an organizational choice based on the distribution and availability of the raw materials on the landscape, as relatively distant exposures or as easily accessed cobbles in paleo-channels, for the glassy ignimbrite and basalt, respectively. Nevertheless, the presence of other raw materials opens the possibility that the different raw materials in the percussive tool assemblage were selected in response to different needs (different contact materials or actions) that required specific raw material properties. The size of raw material ‘packages’ and their shapes (e.g., basalt cobbles for hammerstones vs. large flakes or flat core fragments for anvils) may have been one such consideration.

Against the background of the analytical results, the differential use of raw materials underscores the nuanced nature of the selection criteria applied by the Acheulean tool-makers, with broader implications for important questions about early hominin pre-planning, spatial cognition and behavioural flexibility. Being aware of the limitations of mechanical experiments as a stand-alone method to support detailed use-wear analyses, ‘actualistic experiments’ (i.e., use of the various materials as percussive elements under less controlled conditions) and quantification of mechanical properties of archaeological specimens are the next necessary steps in exploring such questions more comprehensively. These ‘3^rd^ generations experiments’ (*sensu* Marreiros et al. 2020) will help to provide meaningful tests of the created models against the archaeological record.

## 6. Conclusion

This paper presents and discusses an investigation focusing on the characterization of the properties of lithic raw materials that were selected and used by Early Pleistocene Acheulian tool-makers in the Melka Wakena site-complex in the Ethiopian highlands. Using a multidisciplinary approach that includes raw material properties analyses, imaging, and a dedicated experimental program, we were able to show that different raw materials preserve a different damage signature even when submitted to the exact same stress in a controlled and systematized activity.

This paper represents one of the first steps to explore the selection and use of raw materials for percussive activities in Melka Wakena. The data presented here provide a better, quantifiable, and measurable view of raw material variability present at the MW sites. Drawing from our data and observations, we emphasize the importance of preliminary raw material characterization before conducting detailed use-wear analyses on artefacts.

The results of the controlled experiment now allow us to formulate the first set of explanatory hypotheses about the choices of raw material for the percussive tools, which can be explored both through further steps on the study’s replicative experiments and on the archaeological objects. Based on these data, we hypothesize that the raw material selection observed in the archaeological case study may reflect a recognition by early hominins that specific raw materials, even when similar to the naked eye, had particular physical properties that could be ‘tailored’ to particular activities carried out on particular materials. This hypothesis can now be tested with further controlled and manual experiments, varying the object materials (e.g., stone flaking, bone breaking, meat softening etc.), and by direct observation on the archaeological materials.

Our method of analysis provides objective, replicable quantitative results that can be used to improve both intra and inter-site comparisons in the future when more case studies follow this type of workflow to explore percussive tool use. As a second step in this multi-phased research, the raw material characterization data must be analyzed in tandem with additional (mechanized and manual) experimental data and results from use-wear analyses on the archaeological assemblages. The experimental data will become crucial in the course of further functional and technological analyses, not only for the research at MW but also in other regions with the presence of artefacts made of volcanic raw materials.

## Supporting information

S1 File Equipment and settings. (PDF)

S2 File Simplified workflow for 3D model analysis. (PDF)

S3 File Plots hardness, density and volume loss data. (HTML)

S4 File Absolute and relative frequencies of some artifact categories (per raw materials) recovered from excavation at MW2 and MW5 localities. (PDF)

## References

[1] Biro D, Carvalho S, Matsuzawa T. Tools, Traditions, and Technologies: Interdisciplinary Approaches to Chimpanzee Nut Cracking. In: The Mind of the Chimpanzee: Ecological and Experimental Perspectives. University of Chicago Press; 2010. p. 141–55.

[2] Braun DR, Plummer TW, Ditchfield PW, Bishop LC, Ferraro JV. Oldowan Technology and Raw Material Variability at Kanjera South. In: Hovers E, Braun DR, editors. Interdisciplinary Approaches to the Oldowan. Dordrecht: Springer Netherlands; 2009. p. 99–110. (Vertebrate Paleobiology and Paleoanthropology). Available from: 10.1007/978-1-4020-9060-8_9

[3] Carvalho S, Cunha E, Sousa C, Matsuzawa T. Chaînes opératoires and resource-exploitation strategies in chimpanzee (Pan troglodytes) nut cracking. J Hum Evol. 2008;55(1):148–63. 18359504 10.1016/j.jhevol.2008.02.005

[4] Falótico T, Ottoni EB. The manifold use of pounding stone tools by wild capuchin monkeys of Serra da Capivara National Park, Brazil. Behaviour. 2016;153(4):421–42.

[5] Luncz LV, Gill M, Proffitt T, Svensson MS, Kulik L, Malaivijitnond S. Group-specific archaeological signatures of stone tool use in wild macaques. eLife. 2019 Oct 22;8:e46961. doi: 10.7554/eLife.46961 31635691 PMC6805154

[6] Proffitt T, Luncz VL, Malaivijitnond S, Gumert M, Svensson MS, Haslam M. Analysis of wild macaque stone tools used to crack oil palm nuts. R Soc Open Sci. 2018 Mar;5(3):171904. doi: 10.1098/rsos.171904 29657792 PMC5882716

[7] Andrefsky W. The Analysis of Stone Tool Procurement, Production, and Maintenance. J Archaeol Res. 2009;17(1):65–103.

[8] Brantingham PJ. A Neutral Model of Stone Raw Material Procurement. Am Antiq. 2003 Jul;68(3):487–509.

[9] Braun DR, Plummer T, Ferraro JV, Ditchfield P, Bishop LC. Raw material quality and Oldowan hominin toolstone preferences: evidence from Kanjera South, Kenya. J Archaeol Sci. 2009 Jul;36(7):1605–14.

[10] Caruana MV, Tasker D, Stratford DJ. Identifying Raw Material Transportation and Reduction Strategies from the Lithic Scatters at Elandsdrift Farm (Cradle of Humankind World Heritage Site), South Africa. Afr Archaeol Rev. 2019 Jun 1;36(2):271–89.

[11] Delagnes A, Brenet M, Gravina B, Santos F. Exploring the relative influence of raw materials, percussion techniques, and hominin skill levels on the diversity of the early Oldowan assemblages: Insights from the Shungura Formation, Lower Omo Valley, Ethiopia. Petraglia MD, editor. PLOS ONE. 2023 Apr 5;18(4):e0283250. doi: 10.1371/journal.pone.0283250 37018222 PMC10075482

[12] Ekshtain R, Ilani S, Segal I, Hovers E. Local and Nonlocal Procurement of Raw Material in Amud Cave, Israel: The Complex Mobility of Late Middle Paleolithic Groups. Geoarchaeology. 2017;32(2):189–214.

[13] Ekshtain R, Zaidner Y. Raw material exploitation at the Middle Paleolithic site of Nesher Ramla, Israel. Quat Int. 2022 Jun 30;624:34–48.

[14] Eren MI, Roos CI, Story BA, von Cramon-Taubadel N, Lycett SJ. The role of raw material differences in stone tool shape variation: an experimental assessment. J Archaeol Sci. 2014 Sep;49:472–87.

[15] Frahm E, Kandel AW, Gasparyan B. Upper Palaeolithic Settlement and Mobility in the Armenian Highlands: Agent-Based Modeling, Obsidian Sourcing, and Lithic Analysis at Aghitu-3 Cave. J Paleolit Archaeol. 2019 Dec 1;2(4):418–65.

[16] Goldman-Neuman T, Hovers E. Raw material selectivity in Late Pliocene Oldowan sites in the Makaamitalu Basin, Hadar, Ethiopia. J Hum Evol. 2012 Mar 1;62(3):353–66. doi: 10.1016/j.jhevol.2011.05.006 21741072

[17] Goldman-Neuman T, Hovers E. Methodological Considerations in the Study of Oldowan Raw Material Selectivity: Insights from A. L. 894 (Hadar, Ethiopia). In: Hovers E, Braun DR, editors. Interdisciplinary Approaches to the Oldowan. Dordrecht: Springer Netherlands; 2009. p. 71–84. (Vertebrate Paleobiology and Paleoanthropology). Available from: 10.1007/978-1-4020-9060-8_7

[18] Harmand S. Variability in Raw Material Selectivity at the Late Pliocene sites of Lokalalei, West Turkana, Kenya. In: Hovers E, Braun DR, editors. Interdisciplinary Approaches to the Oldowan. Dordrecht: Springer Netherlands; 2009. p. 85–97. (Vertebrate Paleobiology and Paleoanthropology). Available from: 10.1007/978-1-4020-9060-8_8

[19] Pargeter J, de la Peña P, Eren MI. Assessing raw material’s role in bipolar and freehand miniaturized flake shape, technological structure, and fragmentation rates. Archaeol Anthropol Sci. 2019 Nov 1;11(11):5893–907.

[20] Proffitt T. Is there a Developed Oldowan A at Olduvai Gorge? A diachronic analysis of the Oldowan in Bed I and Lower-Middle Bed II at Olduvai Gorge, Tanzania. J Hum Evol. 2018 Jul;120:92–113. doi: 10.1016/j.jhevol.2018.01.006 29752004

[21] Santonja M, Panera J, Rubio-Jara S, Pérez-González A, Uribelarrea D, Domínguez-Rodrigo M, et al. Technological strategies and the economy of raw materials in the TK (Thiongo Korongo) lower occupation, Bed II, Olduvai Gorge, Tanzania. Quat Int. 2014 Feb 16;322–323:181–208.

[22] Webb J, Domanski M. The relationship between lithology, flaking properties and artefact manufacture for Australian silcretes. Archaeometry. 2008 Mar 6;50:555–75.

[23] Proffitt T, Bargalló A, de la Torre I. The Effect of Raw Material on the Identification of Knapping Skill: a Case Study from Olduvai Gorge, Tanzania. J Archaeol Method Theory. 2022 Mar 1;29(1):50–82.

[24] Caruana MV. Exploring the Influence of Predation Risks on Oldowan Tool Use in South Africa. J Field Archaeol. 2020 Nov 16;45(8):608–20.

[25] Caruana MV, Mtshali SP. Assessing the Durability of Oldowan Stone Tools in South Africa: Implications for Quartz Selectivity and Use. Lithic Technol. 2018 Oct 2;43(4):245–54.

[26] Lerner H, Du X, Costopoulos A, Ostoja-Starzewski M. Lithic raw material physical properties and use-wear accrual. J Archaeol Sci. 2007 May;34(5):711–22.

[27] McHenry LJ, de la Torre I. Hominin raw material procurement in the Oldowan-Acheulean transition at Olduvai Gorge. J Hum Evol. 2018 Jul 1;120:378–401. doi: 10.1016/j.jhevol.2017.11.010 29331229

[28] Arroyo A, Harmand S, Roche H, Taylor N. Searching for hidden activities: Percussive tools from the Oldowan and Acheulean of West Turkana, Kenya (2.3–1.76 Ma). J Archaeol Sci. 2020 Nov;123:105238.

[29] Benito-Calvo A, Arroyo A, Sánchez-Romero L, Pante M, de la Torre I. Quantifying 3D Micro-Surface Changes on Experimental Stones Used to Break Bones and Their Implications for the Analysis of Early Stone Age Pounding Tools. Archaeometry. 2018 Jun;60(3):419–36.

[30] Columbu S, Fancello D, Gallello G, Ramacciotti M, Diez-Castillo A. Multi-Analytical Techniques to Define the Mineralogical and Petrophysical Characteristics and Provenance of Siliceous Lithic Findings: The Case Study of La Calvera Rock Shelter (Cantabria, Spain). Minerals. 2023 May;13(5):666.

[31] Reedy CL. Thin-Section Petrography in Studies of Cultural Materials. J Am Inst Conserv. 1994;33(2):115–29.

[32] Rodriguez A, Yanamandra K, Witek L, Wang Z, Behera RK, Iovita R. The effect of worked material hardness on stone tool wear. 2021 Jan 17; 10.1371/journal.pone.0276166 PMC9584531 36264949

[33] Matthews JA, Winkler S. Schmidt-hammer exposure-age dating: a review of principles and practice. Earth-Sci Rev. 2022 Jul 1;230:104038.

[34] Schunk L, Gneisinger W, Calandra I, Marreiros J. The role of artificial contact materials in experimental use-wear studies: A controlled proxy to understand use-wear polish formation. J Archaeol Sci Rep. 2023 Feb;47:103737.

[35] Grosman L, Muller A, Dag I, Goldgeier H, Harush O, Herzlinger G, et al. Artifact3-D: New software for accurate, objective and efficient 3D analysis and documentation of archaeological artifacts. PLOS ONE. 2022 Jun 16;17(6):e0268401. doi: 10.1371/journal.pone.0268401 35709137 PMC9202890

[36] White MA, Campione NE. A three-dimensional approach to visualize pairwise morphological variation and its application to fragmentary palaeontological specimens. PeerJ. 2021 Jan 19;9:e10545. doi: 10.7717/peerj.10545 33552712 PMC7821773

[37] Giusca C, Evans A, Macdonald D, Leach R. The effect of use duration on surface roughness measurements of stone tools. 2011.

[38] Marreiros J, Calandra I, Gneisinger W, Paixão E, Pedergnana A, Schunk L. Rethinking Use-Wear Analysis and Experimentation as Applied to the Study of Past Hominin Tool Use. J Paleolit Archaeol. 2020 May 4;

[39] Paixão E, Pedergnana A, Marreiros J, Dubreuil L, Prévost M, Zaidner Y, et al. Using mechanical experiments to study ground stone tool use: Exploring the formation of percussive and grinding wear traces on limestone tools. J Archaeol Sci Rep. 2021 Jun;37:102971.

[40] Delgado-Raack S, Gómez-Gras D, Risch R. The mechanical properties of macrolithic artifacts: a methodological background for functional analysis. J Archaeol Sci. 2009 Sep;36(9):1823–31.

[41] Iovita R, Schönekeß H, Gaudzinski-Windheuser S, Jäger F. Projectile impact fractures and launching mechanisms: Results of a controlled ballistic experiment using replica Levallois points. J Archaeol Sci. 2014;48(1):73–83.

[42] Key A, Proffitt T, Torre I de la. Raw material optimization and stone tool engineering in the Early Stone Age of Olduvai Gorge (Tanzania). J R Soc Interface. 2020;17(162). doi: 10.1098/rsif.2019.0377 31910772 PMC7014806

[43] Pfleging J, Iovita R, Buchli J. Influence of force and duration on stone tool wear: results from experiments with a force-controlled robot. Archaeol Anthropol Sci. 2019 Nov 1;11(11):5921–35.

[44] Nora DAC. The role of lithic raw materials on tool performance and use: 2021;

[45] Key A, Bartkowiak T, Macdonald DA, Mietlinski P, Gapinski B, de la Torre I, et al. Quantifying Edge Sharpness on Stone Flakes: Comparing Mechanical and Micro-Geometric Definitions Across Multiple Raw Materials from Olduvai Gorge (Tanzania). J Archaeol Method Theory. 2024 Mar 1;31(1):51–74.

[46] Coles JM. Experimental archaeology. London, UK: Academic Press; 1979.

[47] Pereira T, Marreiros J, Paixao E, Martins R. Mechanical experiments to test quartzite vs chert edge reduction. Exploit Raw Mater Prehistory Sourc Process Distrib. 2017;(December 2019):613–26.

[48] Gürbüz RB, Lycett SJ. Could woodworking have influenced variation in the form of Acheulean handaxes? Archaeometry. 2023;65(5):1090–107.

[49] Lewis AR, Williams JC, Buchanan B, Walker RS, Eren MI, Bebber MR. Knapping quality of local versus exotic Upper Mercer chert (Ohio, USA) during the Holocene. Geoarchaeology. 2022;37(3):486–96.

[50] Williams JC, Simone DM, Buchanan B, Boulanger MT, Bebber MR, Eren MI. Nine-thousand years of optimal toolstone selection through the North American Holocene. Antiquity. 2019 Apr;93(368):313–24.

[51] Thompson JC, Carvalho S, Marean CW, Alemseged Z. Origins of the Human Predatory Pattern: The Transition to Large-Animal Exploitation by Early Hominins. Curr Anthropol. 2019 Feb 2;60(1):1–23.

[52] Harmand S, Lewis JE, Feibel CS, Lepre CJ, Prat S, Lenoble A, et al. 3.3-million-year-old stone tools from Lomekwi 3, West Turkana, Kenya. Nature. 2015 May;521(7552):310–5. doi: 10.1038/nature14464 25993961

[53] Hassan A, Sandanger TorkjelM, Brustad M. Level of selected nutrients in meat, liver, tallow and bone marrow from semi-domesticated reindeer (*Rangifer t*. *tarandus L*.). Int J Circumpolar Health. 2012 Jan 31;71(1):17997. 22456051 10.3402/ijch.v71i0.17997 PMC3417664

[54] Arroyo A, de la Torre I. Assessing the function of pounding tools in the Early Stone Age: A microscopic approach to the analysis of percussive artefacts from Beds I and II, Olduvai Gorge (Tanzania). J Archaeol Sci. 2016 Oct 1;74:23–34.

[55] Cabanès J, Borel A, Preysler JB, Lourdeau A, Moncel MH. Palaeolithic polyhedrons, spheroids and bolas over time and space. PLOS ONE. 2022 Jul 28;17(7):e0272135. doi: 10.1371/journal.pone.0272135 35901051 PMC9333226

[56] Goren-Inbar N, Sharon G, Alperson-Afil N, Herzlinger G. A new type of anvil in the Acheulian of Gesher Benot Ya‘aqov, Israel. Philos Trans R Soc B Biol Sci. 2015 Nov 19;370(1682):20140353. doi: 10.1098/rstb.2014.0353 26483531 PMC4614716

[57] Titton S, Barsky D, Bargallo A, Vergès JM, Guardiola M, Solano JG, et al. Active percussion tools from the Oldowan site of Barranco León (Orce, Andalusia, Spain): The fundamental role of pounding activities in hominin lifeways. J Archaeol Sci. 2018 Aug 1;96:131–47.

[58] Hovers E. Chapter 5 ‐ Invention, Reinvention and Innovation: The Makings of Oldowan Lithic Technology. In: Elias S, editor. Developments in Quaternary Sciences. Elsevier; 2012. p. 51–68. (Origins of Human Innovation and Creativity; vol. 16). Available from: https://www.sciencedirect.com/science/article/pii/B9780444538215000051

[59] Toth N, Schick K. An overview of the cognitive implications of the Oldowan Industrial Complex. Azania Archaeol Res Afr. 2018 Jan 2;53(1):3–39.

[60] Beyene Y, Katoh S, WoldeGabriel G, Hart WK, Uto K, Sudo M, et al. The characteristics and chronology of the earliest Acheulean at Konso, Ethiopia. Proc Natl Acad Sci. 2013 Jan 29;110(5):1584. doi: 10.1073/pnas.1221285110 23359714 PMC3562807

[61] Diez-Martín F, Sánchez Yustos P, Uribelarrea D, Baquedano E, Mark DF, Mabulla A, et al. The Origin of The Acheulean: The 1.7 Million-Year-Old Site of FLK West, Olduvai Gorge (Tanzania). Sci Rep. 2015 Dec 7;5(1):17839. doi: 10.1038/srep17839 26639785 PMC4671088

[62] Lepre C, Roche H, Harmand S, Quinn R, Brugal JP, Texier PJ, et al. An earlier origin for the Acheulian. Nature. 2011 Sep 1;477:82–5. doi: 10.1038/nature10372 21886161

[63] Gallotti R, Mussi M. The Emergence of the Acheulean in East Africa: Historical Perspectives and Current Issues. In: Gallotti R, Mussi M, editors. The Emergence of the Acheulean in East Africa and Beyond: Contributions in Honor of Jean Chavaillon. Cham: Springer International Publishing; 2018. p. 1–12. (Vertebrate Paleobiology and Paleoanthropology). Available from: 10.1007/978-3-319-75985-2_1

[64] Bar-Yosef O, Goren-Inbar N. The Lithic Assemblages of ‘Ubeidiya: A Lower Palaeolithic Site in the Jordan Valley. Qedem. 1993;34:III–266.

[65] Favreau J. Sourcing Oldowan and Acheulean stone tools in Eastern Africa: Aims, methods, challenges, and state of knowledge. Quat Sci Adv. 2023 Jan 1;9:100068.

[66] Gossa T, Hovers E. Continuity and change in lithic techno-economy of the early Acheulian on the Ethiopian highland: A case study from locality MW2; the Melka Wakena site-complex. Petraglia MD, editor. PLOS ONE. 2022 Dec 7;17(12):e0277029. doi: 10.1371/journal.pone.0277029 36477016 PMC9728887

[67] Hovers E, Gossa T, Asrat A, Niespolo EM, Resom A, Renne PR, et al. The expansion of the Acheulian to the Southeastern Ethiopian Highlands: Insights from the new early Pleistocene site-complex of Melka Wakena. Quat Sci Rev. 2021 Feb 1;253:106763.

[68] Gossa T, Hovers E. The large flake-based Acheulian technology at the Melka Wakena site complex: the case of locality MW5. Archaeol Anthropol Sci. accepted;

[69] Gossa T. The Melka Wakena site complex, south-central Ethiopia: Lithic Technology, Raw material economy, and Regional perspectives on the early Acheulian on the Ethiopian Highlands. [PhD thesis]. Jerusalem: The Hebrew University of Jerusalem; 2020.

[70] Resom A, Asrat A, Gossa T, Hovers E. Petrogenesis and depositional history of felsic pyroclastic rocks from the Melka Wakena archaeological site-complex in South central Ethiopia. J Afr Earth Sci. 2018 Jun;142:93–111.

[71] Resom A. Petrogenetic evolution of the melka wakena pyroclastic deposits: implications for the depositional history of the intercalated volcano- sedimentary rocks. [Addis Ababa]: School of Earth Sciences of the Addis Ababa; 2017.

[72] Calandra I, Gneisinger W, Marreiros J. A versatile mechanized setup for controlled experiments in archeology. STAR Sci Technol Archaeol Res. 2020 Jan 1;6(1):30–40.

[73] Rockafellar RT, Wets RJB, Wets M. Variational Analysis. Corrected 1998, edition. Berlin: Springer; 1997. 733 p.

