# Supplementary material for: Exploring early Acheulian technological decision-making: A controlled experimental approach to raw material selection for percussive artifacts in Melka Wakena, Ethiopia
Source: PLoS One. 2025 Jan 9;20(1):e0314039. doi: 10.1371/journal.pone.0314039 (PMC11717217; doi:10.1371/journal.pone.0314039)
Supplement: S1 File — (PDF) [file pone.0314039.s001.pdf]

SOM 1

Equipment and settings:

Imaging technique	***Equipment***	***Objective***	**Resolution**	**Acquisition method**	**Software version**
**Photography**	Nikon DSLR D160	Nikon AF-S VR Micro-Nikkor 105 mm f/2.8G IF-ED			
**3D scanning**	3D structured-light scanner, Aicon SmartScan-HE R8 (now part of Hexagon AB)		Field of View: 110 × 80 × 70mm , 33 µm point-to-point distance	structured-light, ~16 scans on automatic turn table	OptoCat 2018R1
**3D digital microscopy**	ZEISS Smartzoom 5	PlanApo D 1.6×/NA 0.1,WD 36 mm	Field of View: 10.475 × 7.856 mm (34x)	White LED, reflected ringlight, EDF / Stitching	Smartzoom software with Shuttle&Find module
**CT-scannig***	Phoenix V|tome|x L				

*More detail about setting on https://zenodo.org/records/10631628
