# Supplementary material for: Exploring early Acheulian technological decision-making: A controlled experimental approach to raw material selection for percussive artifacts in Melka Wakena, Ethiopia
Source: PLoS One. 2025 Jan 9;20(1):e0314039. doi: 10.1371/journal.pone.0314039 (PMC11717217; doi:10.1371/journal.pone.0314039)
Supplement: S2 File — (PDF) [file pone.0314039.s002.pdf]

SOM 2 (Simplified workflow for 3D model analysis used in Paixão et al. 2024)

This workflow aims to show the step-by-step method to quantify the surface alterations between 3D models, representing the *before* and *after* steps in our experiments, using the software *CloudCompare Version 2.12.3(Kyiv)*. The workflow for 3D model comparison is adapted from (White and Campione 2021; Nora 2021)

1. Import
    a. Import both models (*before* and *after*) as stl. format into the *CloudCompare.*

2. Alignment
    a. Select both models and align the model using the pair-picking method (use at least 3 common points) and click on align (tools -> Registration -> Align (point pairs picking)).
    b. Select both models and apply the fine registration to improve the alignment (tools -> Registration -> Fine registration (ICP)).
3. Calculate distances
    a. Compute the Cloud-Mesh distance (tools -> Distances -> Could/Mesh Dist)
    b. In the computed mesh properties menu verify the "steps" (256 is the default division of the model to calculate the distances)
4. Visualize data
    a. In the toolbar click on Show Histogram (normally locater in the left toolbar menu)
5. Export data
    a. In the visualization menu of the histogram, click on export as csv.

Rerefences:

Nora, David. 2021, 'The role of lithic raw materials on tool performance and use: The efficiency and durability on stone tools edge. 'M.A. Thesis. University of Algarve. Faro.

White, Matt A., and Nicolás E. Campione. 2021. 'A Three-Dimensional Approach to Visualize Pairwise Morphological Variation and Its Application to Fragmentary Palaeontological Specimens'. *PeerJ* 9 (January): e10545. https://doi.org/10.7717/peerj.10545.
