# Supplementary material for: Exploring early Acheulian technological decision-making: A controlled experimental approach to raw material selection for percussive artifacts in Melka Wakena, Ethiopia
Source: PLoS One. 2025 Jan 9;20(1):e0314039. doi: 10.1371/journal.pone.0314039 (PMC11717217; doi:10.1371/journal.pone.0314039)
Supplement: S3 File — (HTML) [file pone.0314039.s003.html]

Plots Hardness, density and volume loss data


# Plots Hardness, density and volume loss data

#### Eduardo Paixão, Tegenu Gossa, Walter Gneisinger, João Marreiros, Sören Tholen, Ivan Calandra, Erella Hovers

#### 2024-02-07 16:19:16.064531

---

# Content

This script reads and plots the data from the 3D volume loss and Leeb
Rebound hardness. 3D volume loss was calculated using CloudCompare
cloud-to-mesh distance tool. Hardness was measured on each rock using
the device Equotip 550 Leeb Impact device C (HLC). For details on the
methods and data acquisition, please visit the Materials and Methods
section of the paper.

The knit directory for this script is the project directory.

---

# Load packages

```
library(R.utils)
```

```
## Warning: package 'R.utils' was built under R version 4.3.1
```

```
## Loading required package: R.oo
```

```
## Loading required package: R.methodsS3
```

```
## R.methodsS3 v1.8.2 (2022-06-13 22:00:14 UTC) successfully loaded. See ?R.methodsS3 for help.
```

```
## R.oo v1.25.0 (2022-06-12 02:20:02 UTC) successfully loaded. See ?R.oo for help.
```

```
## 
## Attaching package: 'R.oo'
```

```
## The following object is masked from 'package:R.methodsS3':
## 
##     throw
```

```
## The following objects are masked from 'package:methods':
## 
##     getClasses, getMethods
```

```
## The following objects are masked from 'package:base':
## 
##     attach, detach, load, save
```

```
## R.utils v2.12.3 (2023-11-18 01:00:02 UTC) successfully loaded. See ?R.utils for help.
```

```
## 
## Attaching package: 'R.utils'
```

```
## The following object is masked from 'package:utils':
## 
##     timestamp
```

```
## The following objects are masked from 'package:base':
## 
##     cat, commandArgs, getOption, isOpen, nullfile, parse, warnings
```

```
library(ggplot2)
```

```
## Warning: package 'ggplot2' was built under R version 4.3.1
```

```
library(tools)
library(tidyverse)
```

```
## Warning: package 'readr' was built under R version 4.3.1
```

```
## Warning: package 'dplyr' was built under R version 4.3.1
```

```
## Warning: package 'stringr' was built under R version 4.3.1
```

```
## Warning: package 'lubridate' was built under R version 4.3.1
```

```
## ── Attaching core tidyverse packages ──────────────────────── tidyverse 2.0.0 ──
## ✔ dplyr     1.1.4     ✔ readr     2.1.5
## ✔ forcats   1.0.0     ✔ stringr   1.5.1
## ✔ lubridate 1.9.3     ✔ tibble    3.2.1
## ✔ purrr     1.0.2     ✔ tidyr     1.3.0
```

```
## ── Conflicts ────────────────────────────────────────── tidyverse_conflicts() ──
## ✖ tidyr::extract() masks R.utils::extract()
## ✖ dplyr::filter()  masks stats::filter()
## ✖ dplyr::lag()     masks stats::lag()
## ℹ Use the conflicted package (<http://conflicted.r-lib.org/>) to force all conflicts to become errors
```

```
library(doBy)
```

```
## Warning: package 'doBy' was built under R version 4.3.1
```

```
## 
## Attaching package: 'doBy'
## 
## The following object is masked from 'package:dplyr':
## 
##     order_by
```

```
library(ggrepel)
```

```
## Warning: package 'ggrepel' was built under R version 4.3.1
```

```
library(flextable)
```

```
## Warning: package 'flextable' was built under R version 4.3.1
```

```
## 
## Attaching package: 'flextable'
## 
## The following object is masked from 'package:purrr':
## 
##     compose
```

```
library(readr)
library(ggpubr)
```

```
## 
## Attaching package: 'ggpubr'
## 
## The following objects are masked from 'package:flextable':
## 
##     border, font, rotate
```

---

# Import and preview data

```
# Data on hardness, density and volume loss are imported individually

hlcdata <- read_csv2("../rawdata/hlcdata.csv")
```

```
## ℹ Using "','" as decimal and "'.'" as grouping mark. Use `read_delim()` for more control.
```

```
## Rows: 5 Columns: 30
## ── Column specification ────────────────────────────────────────────────────────
## Delimiter: ";"
## chr  (11): cubeid, Date, Probe, Probe serial, Probe verification, Operator, ...
## dbl  (17): Upper limit, Lower limit, Readings, Mean, Min, Max, Range, readin...
## num   (1): Std. dev.
## time  (1): Time
## 
## ℹ Use `spec()` to retrieve the full column specification for this data.
## ℹ Specify the column types or set `show_col_types = FALSE` to quiet this message.
```

```
volumelossdata2 <- read_csv2("../rawdata/volumelossdata.csv")
```

```
## ℹ Using "','" as decimal and "'.'" as grouping mark. Use `read_delim()` for more control.
## Rows: 5120 Columns: 8── Column specification ────────────────────────────────────────────────────────
## Delimiter: ";"
## chr (6): cubeid, Sample, Material, Cycle, Class start, Class end
## dbl (2): Class, Value
## ℹ Use `spec()` to retrieve the full column specification for this data.
## ℹ Specify the column types or set `show_col_types = FALSE` to quiet this message.
```

```
densitydata <- read_csv2("../rawdata/densitydata.csv")
```

```
## ℹ Using "','" as decimal and "'.'" as grouping mark. Use `read_delim()` for more control.
## Rows: 5 Columns: 5── Column specification ────────────────────────────────────────────────────────
## Delimiter: ";"
## chr (2): cubeid, Material
## dbl (3): mass, volume, density
## ℹ Use `spec()` to retrieve the full column specification for this data.
## ℹ Specify the column types or set `show_col_types = FALSE` to quiet this message.
```

```
str(hlcdata)
```

```
## spc_tbl_ [5 × 30] (S3: spec_tbl_df/tbl_df/tbl/data.frame)
##  $ cubeid             : chr [1:5] "DW-G1-S3" "DW-G3-S3" "DW-G4-S3" "KW-G1-S3" ...
##  $ Date               : chr [1:5] "16/11/2022" "16/11/2022" "16/11/2022" "16/11/2022" ...
##  $ Time               : 'hms' num [1:5] 15:55:29 15:47:15 15:52:31 15:50:56 ...
##   ..- attr(*, "units")= chr "secs"
##  $ Probe              : chr [1:5] "Equotip Leeb Impact Device C" "Equotip Leeb Impact Device C" "Equotip Leeb Impact Device C" "Equotip Leeb Impact Device C" ...
##  $ Probe serial       : chr [1:5] "IC51-004-0185" "IC51-004-0185" "IC51-004-0185" "IC51-004-0185" ...
##  $ Probe verification : chr [1:5] "--" "--" "--" "--" ...
##  $ Operator           : chr [1:5] "Paixao" "Paixao" "Paixao" "Paixao" ...
##  $ Device serial      : chr [1:5] "UP01-003-1680" "UP01-003-1680" "UP01-003-1680" "UP01-003-1680" ...
##  $ Direction (Equotip): chr [1:5] "Automatic" "Automatic" "Automatic" "Automatic" ...
##  $ Material           : chr [1:5] "basalt" "scoria" "pumaceousignimbrite" "glassyignimbrite" ...
##  $ Conversion Std.    : chr [1:5] "Default" "Default" "Default" "Default" ...
##  $ Scale              : chr [1:5] "HLC" "HLC" "HLC" "HLC" ...
##  $ Upper limit        : num [1:5] 528 528 528 528 528
##  $ Lower limit        : num [1:5] 498 498 498 498 498
##  $ Readings           : num [1:5] 10 10 10 10 10
##  $ Mean               : num [1:5] 769 717 528 907 672
##  $ Min                : num [1:5] 743 604 456 789 591
##  $ Max                : num [1:5] 813 834 605 938 741
##  $ Range              : num [1:5] 70 229 149 149 149
##  $ Std. dev.          : num [1:5] 23 86 455 412 445
##  $ reading1           : num [1:5] 769 644 500 907 591
##  $ reading2           : num [1:5] 750 688 600 920 609
##  $ reading3           : num [1:5] 750 834 456 920 690
##  $ reading4           : num [1:5] 743 824 518 888 671
##  $ reading5           : num [1:5] 783 653 511 921 654
##  $ reading6           : num [1:5] 813 614 488 925 741
##  $ reading7           : num [1:5] 747 701 509 925 718
##  $ reading8           : num [1:5] 800 604 605 789 707
##  $ reading9           : num [1:5] 755 810 532 932 651
##  $ reading10          : num [1:5] 782 797 562 938 685
##  - attr(*, "spec")=
##   .. cols(
##   ..   cubeid = col_character(),
##   ..   Date = col_character(),
##   ..   Time = col_time(format = ""),
##   ..   Probe = col_character(),
##   ..   `Probe serial` = col_character(),
##   ..   `Probe verification` = col_character(),
##   ..   Operator = col_character(),
##   ..   `Device serial` = col_character(),
##   ..   `Direction (Equotip)` = col_character(),
##   ..   Material = col_character(),
##   ..   `Conversion Std.` = col_character(),
##   ..   Scale = col_character(),
##   ..   `Upper limit` = col_double(),
##   ..   `Lower limit` = col_double(),
##   ..   Readings = col_double(),
##   ..   Mean = col_double(),
##   ..   Min = col_double(),
##   ..   Max = col_double(),
##   ..   Range = col_double(),
##   ..   `Std. dev.` = col_number(),
##   ..   reading1 = col_double(),
##   ..   reading2 = col_double(),
##   ..   reading3 = col_double(),
##   ..   reading4 = col_double(),
##   ..   reading5 = col_double(),
##   ..   reading6 = col_double(),
##   ..   reading7 = col_double(),
##   ..   reading8 = col_double(),
##   ..   reading9 = col_double(),
##   ..   reading10 = col_double()
##   .. )
##  - attr(*, "problems")=<externalptr>
```

```
str(volumelossdata2)
```

```
## spc_tbl_ [5,120 × 8] (S3: spec_tbl_df/tbl_df/tbl/data.frame)
##  $ cubeid     : chr [1:5120] "DW-G1-S3" "DW-G1-S3" "DW-G1-S3" "DW-G1-S3" ...
##  $ Sample     : chr [1:5120] "DW-G1-S3-V1" "DW-G1-S3-V1" "DW-G1-S3-V1" "DW-G1-S3-V1" ...
##  $ Material   : chr [1:5120] "basalt" "basalt" "basalt" "basalt" ...
##  $ Cycle      : chr [1:5120] "0-1" "0-1" "0-1" "0-1" ...
##  $ Class      : num [1:5120] 1 2 3 4 5 6 7 8 9 10 ...
##  $ Value      : num [1:5120] 2 4 3 0 1 0 3 0 2 2 ...
##  $ Class start: chr [1:5120] "0.500000000000" "0.500338319922" "0.500676639844" "0.501014959766" ...
##  $ Class end  : chr [1:5120] "0.500338319922" "0.500676639844" "0.501014959766" "0.501353279687" ...
##  - attr(*, "spec")=
##   .. cols(
##   ..   cubeid = col_character(),
##   ..   Sample = col_character(),
##   ..   Material = col_character(),
##   ..   Cycle = col_character(),
##   ..   Class = col_double(),
##   ..   Value = col_double(),
##   ..   `Class start` = col_character(),
##   ..   `Class end` = col_character()
##   .. )
##  - attr(*, "problems")=<externalptr>
```

```
str(densitydata)
```

```
## spc_tbl_ [5 × 5] (S3: spec_tbl_df/tbl_df/tbl/data.frame)
##  $ cubeid  : chr [1:5] "DW-G1-S3" "KW-G1-S3" "MW6-G2-S3" "DW-G4-S3" ...
##  $ Material: chr [1:5] "basalt" "glassyignimbrite" "ignimbrite" "pumaceousignimbrite" ...
##  $ mass    : num [1:5] 55.2 46.8 39.5 36.3 46.6
##  $ volume  : num [1:5] 15.6 15.6 15.6 15.6 15.6
##  $ density : num [1:5] 3.53 3 2.53 2.32 2.98
##  - attr(*, "spec")=
##   .. cols(
##   ..   cubeid = col_character(),
##   ..   Material = col_character(),
##   ..   mass = col_double(),
##   ..   volume = col_double(),
##   ..   density = col_double()
##   .. )
##  - attr(*, "problems")=<externalptr>
```

# Manipulate hardness data

```
# HLC data, Multiple variables are stored in column names, so transform it into observations per row
hlcdatalong <- hlcdata %>% 
  pivot_longer(c(`reading1`, `reading2`,`reading3`, `reading4`, `reading5`, `reading6`, `reading7`, `reading8`, `reading9`, `reading10`), names_to = "hlcvalues", values_to = "Hardness")

write_csv(hlcdatalong, "../deriveddata/hlcdatalong.csv")
```

# Summarise data

```
# summarise by "Material" but keep other columns

hlcstats <- hlcdatalong %>% group_by(cubeid, Material) %>%
      summarise(
      hlccount = n(),
      hlcmax = max(Hardness, na.rm = TRUE),
      hlcmin = min (Hardness, na.rm = TRUE),
      hlcmean = mean(Hardness, na.rm = TRUE),
      hlcsd = sd(Hardness, na.rm = TRUE),
      hlcmedian = median(Hardness, na.rm = TRUE),
  )
```

```
## `summarise()` has grouped output by 'cubeid'. You can override using the
## `.groups` argument.
```

```
# summarise by "Sample" but keep other columns

volumelossstats <- volumelossdata2 %>% group_by(cubeid, Sample, Material, Cycle) %>%
      summarise(
      vlcount = n(),
      vlmax = max(Value, na.rm = TRUE),
      vlmin = min (Value, na.rm = TRUE),
      vlmean = mean(Value, na.rm = TRUE),
      vlsd = sd(Value, na.rm = TRUE),
      vlmedian = median(Value, na.rm = TRUE),
   )
```

```
## `summarise()` has grouped output by 'cubeid', 'Sample', 'Material'. You can
## override using the `.groups` argument.
```

```
# see data summary
hlcstats
```

```
## # A tibble: 5 × 8
## # Groups:   cubeid [5]
##   cubeid    Material            hlccount hlcmax hlcmin hlcmean hlcsd hlcmedian
##   <chr>     <chr>                  <int>  <dbl>  <dbl>   <dbl> <dbl>     <dbl>
## 1 DW-G1-S3  basalt                    10    813    743    769.  24.4      762 
## 2 DW-G3-S3  scoria                    10    834    604    717.  90.7      694.
## 3 DW-G4-S3  pumaceousignimbrite       10    605    456    528.  47.8      514.
## 4 KW-G1-S3  glassyignimbrite          10    938    789    906.  43.5      920.
## 5 MW6-G2-S3 ignimbrite                10    741    591    672.  47.0      678
```

```
volumelossstats
```

```
## # A tibble: 20 × 10
## # Groups:   cubeid, Sample, Material [20]
##    cubeid    Sample  Material Cycle vlcount vlmax vlmin  vlmean    vlsd vlmedian
##    <chr>     <chr>   <chr>    <chr>   <int> <dbl> <dbl>   <dbl>   <dbl>    <dbl>
##  1 DW-G1-S3  DW-G1-… basalt   0-1       256     5     0 1.14e+0   1.28       1  
##  2 DW-G1-S3  DW-G1-… basalt   0-1       256    11     0 3.79e+0   2.37       3  
##  3 DW-G1-S3  DW-G1-… basalt   0-1       256     2     0 6.64e-2   0.279      0  
##  4 DW-G1-S3  DW-G1-… basalt   0-1       256     5     0 1.30e+0   1.16       1  
##  5 DW-G3-S3  DW-G3-… scoria   0-1       256    23     0 6.01e+0   3.71       5  
##  6 DW-G3-S3  DW-G3-… scoria   0-1       256   115     2 3.98e+1  27.7       34  
##  7 DW-G3-S3  DW-G3-… scoria   0-1       256    57     0 9.43e+0  15.7        1  
##  8 DW-G3-S3  DW-G3-… scoria   0-1       256    50     0 1.54e+1  12.0       12  
##  9 DW-G4-S3  DW-G4-… pumaceo… 0-1       256   563     9 1.74e+2 159.        93.5
## 10 DW-G4-S3  DW-G4-… pumaceo… 0-1       256   474     7 1.54e+2 120.       106. 
## 11 DW-G4-S3  DW-G4-… pumaceo… 0-1       256   463    10 1.61e+2 122.       112  
## 12 DW-G4-S3  DW-G4-… pumaceo… 0-1       256   668     7 1.78e+2 162.       108. 
## 13 KW-G1-S3  KW-G1-… glassyi… 0-1       256     5     0 1.22e+0   1.02       1  
## 14 KW-G1-S3  KW-G1-… glassyi… 0-1       256     0     0 0         0          0  
## 15 KW-G1-S3  KW-G1-… glassyi… 0-1       256     0     0 0         0          0  
## 16 KW-G1-S3  KW-G1-… glassyi… 0-1       256     0     0 0         0          0  
## 17 MW6-G2-S3 MW6-G2… ignimbr… 0-1       256    97     2 2.53e+1  19.1       19  
## 18 MW6-G2-S3 MW6-G2… ignimbr… 0-1       256    39     0 1.93e+1   8.03      20  
## 19 MW6-G2-S3 MW6-G2… ignimbr… 0-1       256   169     6 7.06e+1  40.8       61.5
## 20 MW6-G2-S3 MW6-G2… ignimbr… 0-1       256    96     2 2.60e+1  17.7       23
```

```
# save the results 
write_csv(hlcstats, "../stats/stats_hlc.csv")
write_csv(volumelossstats, "../stats/stats_volumeloss.csv")

# join data tables

hlcdensity_data <- full_join(hlcstats, densitydata,
              by = join_by("cubeid", "Material"), keep = F)

full_data <- full_join(hlcdensity_data, volumelossstats,
              by = join_by("cubeid", "Material"))

# save single dataset
write_csv(full_data, "../deriveddata/full_data.csv")
```

# Plot and explore hardness data, organised by raw material

```
# Boxplot and distribution of all hardness values (10 per each raw material)

hlcplot <- ggplot(hlcdatalong, aes(Material, Hardness, colour = Material)) +
  geom_boxplot() +
  geom_jitter() +
  guides(color = FALSE)  +
  scale_x_discrete(labels = c("Basalt", "Glassy Ignimbrite", "Ignimbrite", "Pumaceous Ignimbrite", "Scoria")) +
  labs(y = "Hardness mean (HLC)", x = "Raw material", colour = "Raw Material")
```

```
## Warning: The `<scale>` argument of `guides()` cannot be `FALSE`. Use "none" instead as
## of ggplot2 3.3.4.
## This warning is displayed once every 8 hours.
## Call `lifecycle::last_lifecycle_warnings()` to see where this warning was
## generated.
```

```
ggsave("../plots/hlcdata.png")
```

```
## Saving 7 x 5 in image
```

```
print(hlcplot)
```

# Plot and explore volume loss data, organised by raw material

```
# boxplot with absolute values for all samples organysed by raw material

volumeplot <- ggplot(full_data, aes(Material, vlmax, colour = Material)) +
  geom_boxplot() +
  geom_jitter() +
  guides(color = FALSE)  +
  scale_x_discrete(labels = c("Basalt", "Glassy Ignimbrite", "Ignimbrite", "Pumaceous Ignimbrite", "Scoria")) +
  labs(y = "Damage (absolute Hausdorff distance, aHd)", x = "Raw material", colour = "Raw Material")

print(volumeplot)
```

```
ggsave("../plots/damage_rawmaterial.png")
```

```
## Saving 7 x 5 in image
```

# Relationship betwen hardness, density, and volume loss

```
# scatterplot to show relationship between variables (dependent, volume loss; and independent, density and hardness)

scat <- ggplot(full_data, aes(hlcmean, vlmax, color = Material)) + 
  geom_point() +
  scale_color_discrete(labels = c("Basalt", "Glassy Ignimbrite", "Ignimbrite", "Pumaceous Ignimbrite", "Scoria")) +
  labs(y = "Damage (absolute Hausdorff distance, aHd)", x = "Hardness mean (HLC)", colour = "Raw Material")

ggsave("../plots/hardvolume.png")
```

```
## Saving 7 x 5 in image
```

```
print(scat)
```

```
scat2 <- ggplot(full_data,aes(density, vlmax, color = Material)) + 
  geom_point() +
  scale_color_discrete(labels = c("Basalt", "Glassy Ignimbrite", "Ignimbrite", "Pumaceous Ignimbrite", "Scoria")) +
  labs(y = "Damage (absolute Hausdorff distance), aHd", x = "Density", colour = "Raw Material")

ggsave("../plots/densityvolume.png")
```

```
## Saving 7 x 5 in image
```

```
print(scat2)
```

```
scat3 <- ggplot(full_data,aes(density, hlcmean, color = Material)) + 
  geom_point()+
  scale_color_discrete(labels = c("Basalt", "Glassy Ignimbrite", "Ignimbrite", "Pumaceous Ignimbrite", "Scoria")) +
  labs(y = "Hardness mean (HLC)", x = "Density", colour = "Raw Material")

ggsave("../plots/densityhard.png")
```

```
## Saving 7 x 5 in image
```

```
print(scat3)
```

```
# combine all data plots and single legend

arranged2 <- ggarrange(scat3, scat, scat2, common.legend = TRUE, ncol = 3, nrow = 1)

print(arranged2)
```

```
ggsave("../plots/arranged2.png")
```

```
## Saving 7 x 5 in image
```

# Bootstrapping

## Bootstrapped Kruskal–Wallis H (p-value, 95% confidence interval) for volume loss (damage) by raw material (more than 3 groups).

```
##   2.5%  97.5% 
## 0.0010 0.0021
```

```
## Warning in wilcox.test.default(xi, xj, paired = paired, ...): cannot compute
## exact p-value with ties

## Warning in wilcox.test.default(xi, xj, paired = paired, ...): cannot compute
## exact p-value with ties

## Warning in wilcox.test.default(xi, xj, paired = paired, ...): cannot compute
## exact p-value with ties

## Warning in wilcox.test.default(xi, xj, paired = paired, ...): cannot compute
## exact p-value with ties

## Warning in wilcox.test.default(xi, xj, paired = paired, ...): cannot compute
## exact p-value with ties

## Warning in wilcox.test.default(xi, xj, paired = paired, ...): cannot compute
## exact p-value with ties

## Warning in wilcox.test.default(xi, xj, paired = paired, ...): cannot compute
## exact p-value with ties
```

```
## 
##  Pairwise comparisons using Wilcoxon rank sum test with continuity correction 
## 
## data:  full_data$vlmax and full_data$Material 
## 
##                     basalt glassyignimbrite ignimbrite pumaceousignimbrite
## glassyignimbrite    0.106  -                -          -                  
## ignimbrite          0.037  0.037            -          -                  
## pumaceousignimbrite 0.037  0.037            0.037      -                  
## scoria              0.037  0.037            0.486      0.037              
## 
## P value adjustment method: BH
```

# Kendall rank correlation between variables

```
## Warning in cor.test.default(full_data$hlcmean, full_data$vlmax, method =
## "kendall"): Cannot compute exact p-value with ties
```

```
## 
##  Kendall's rank correlation tau
## 
## data:  full_data$hlcmean and full_data$vlmax
## z = -4.8658, p-value = 1.14e-06
## alternative hypothesis: true tau is not equal to 0
## sample estimates:
##        tau 
## -0.8509106
```

```
## Warning in cor.test.default(full_data$density, full_data$vlmax, method =
## "kendall"): Cannot compute exact p-value with ties
```

```
## 
##  Kendall's rank correlation tau
## 
## data:  full_data$density and full_data$vlmax
## z = -4.066, p-value = 4.783e-05
## alternative hypothesis: true tau is not equal to 0
## sample estimates:
##        tau 
## -0.7110349
```

# Export manuscript tables

```
# select and format datasets to be included in the paper

## density and hardness data organised by Material ((change column names))
colnames(hlcdensity_data) <- c("Cube ID", "Raw Material", "Hardness (counts)", "Hardness (min)", "Hardness (max)", "Hardness (mean)", "Hardness (median)", "Hardness (sd)", "Mass (gr)", "Volume (cm3)", "Density")

print(hlcdensity_data)
```

```
## # A tibble: 5 × 11
## # Groups:   Cube ID [5]
##   `Cube ID` `Raw Material` `Hardness (counts)` `Hardness (min)` `Hardness (max)`
##   <chr>     <chr>                        <int>            <dbl>            <dbl>
## 1 DW-G1-S3  basalt                          10              813              743
## 2 DW-G3-S3  scoria                          10              834              604
## 3 DW-G4-S3  pumaceousigni…                  10              605              456
## 4 KW-G1-S3  glassyignimbr…                  10              938              789
## 5 MW6-G2-S3 ignimbrite                      10              741              591
## # ℹ 6 more variables: `Hardness (mean)` <dbl>, `Hardness (median)` <dbl>,
## #   `Hardness (sd)` <dbl>, `Mass (gr)` <dbl>, `Volume (cm3)` <dbl>,
## #   Density <dbl>
```

```
write_csv(hlcdensity_data, "../deriveddata/hlcdensity_data.csv")

## Volume loss data organised by Sample (change column names)
colnames(volumelossstats) <- c("Cube ID", "Sample", "Raw material", "Cycle", "Volume loss (counts)", "Volume loss (max)", "Volume loss (min", "Volume loss (mean)", "Volume loss (sd)", "Volume loss (median)")

print(volumelossstats)
```

```
## # A tibble: 20 × 10
## # Groups:   Cube ID, Sample, Raw material [20]
##    `Cube ID` Sample       `Raw material`      Cycle `Volume loss (counts)`
##    <chr>     <chr>        <chr>               <chr>                  <int>
##  1 DW-G1-S3  DW-G1-S3-V1  basalt              0-1                      256
##  2 DW-G1-S3  DW-G1-S3-V2  basalt              0-1                      256
##  3 DW-G1-S3  DW-G1-S3-V3  basalt              0-1                      256
##  4 DW-G1-S3  DW-G1-S3-V4  basalt              0-1                      256
##  5 DW-G3-S3  DW-G3-S3-V1  scoria              0-1                      256
##  6 DW-G3-S3  DW-G3-S3-V2  scoria              0-1                      256
##  7 DW-G3-S3  DW-G3-S3-V3  scoria              0-1                      256
##  8 DW-G3-S3  DW-G3-S3-V4  scoria              0-1                      256
##  9 DW-G4-S3  DW-G4-S3-V1  pumaceousignimbrite 0-1                      256
## 10 DW-G4-S3  DW-G4-S3-V2  pumaceousignimbrite 0-1                      256
## 11 DW-G4-S3  DW-G4-S3-V3  pumaceousignimbrite 0-1                      256
## 12 DW-G4-S3  DW-G4-S3-V4  pumaceousignimbrite 0-1                      256
## 13 KW-G1-S3  KW-G1-S3-V1  glassyignimbrite    0-1                      256
## 14 KW-G1-S3  KW-G1-S3-V2  glassyignimbrite    0-1                      256
## 15 KW-G1-S3  KW-G1-S3-V3  glassyignimbrite    0-1                      256
## 16 KW-G1-S3  KW-G1-S3-V4  glassyignimbrite    0-1                      256
## 17 MW6-G2-S3 MW6-G2-S3-V1 ignimbrite          0-1                      256
## 18 MW6-G2-S3 MW6-G2-S3-V2 ignimbrite          0-1                      256
## 19 MW6-G2-S3 MW6-G2-S3-V3 ignimbrite          0-1                      256
## 20 MW6-G2-S3 MW6-G2-S3-V4 ignimbrite          0-1                      256
## # ℹ 5 more variables: `Volume loss (max)` <dbl>, `Volume loss (min` <dbl>,
## #   `Volume loss (mean)` <dbl>, `Volume loss (sd)` <dbl>,
## #   `Volume loss (median)` <dbl>
```

```
write_csv(volumelossstats, "../deriveddata/volumeloss_stats.csv")
```

---

# sessionInfo() and RStudio version

```
sessionInfo()
```

```
## R version 4.3.0 (2023-04-21)
## Platform: aarch64-apple-darwin20 (64-bit)
## Running under: macOS 14.2.1
## 
## Matrix products: default
## BLAS:   /Library/Frameworks/R.framework/Versions/4.3-arm64/Resources/lib/libRblas.0.dylib 
## LAPACK: /Library/Frameworks/R.framework/Versions/4.3-arm64/Resources/lib/libRlapack.dylib;  LAPACK version 3.11.0
## 
## locale:
## [1] en_US.UTF-8/en_US.UTF-8/en_US.UTF-8/C/en_US.UTF-8/en_US.UTF-8
## 
## time zone: Europe/London
## tzcode source: internal
## 
## attached base packages:
## [1] tools     stats     graphics  grDevices utils     datasets  methods  
## [8] base     
## 
## other attached packages:
##  [1] ggpubr_0.6.0.999  flextable_0.9.4   ggrepel_0.9.5     doBy_4.6.20      
##  [5] lubridate_1.9.3   forcats_1.0.0     stringr_1.5.1     dplyr_1.1.4      
##  [9] purrr_1.0.2       readr_2.1.5       tidyr_1.3.0       tibble_3.2.1     
## [13] tidyverse_2.0.0   ggplot2_3.4.4     R.utils_2.12.3    R.oo_1.25.0      
## [17] R.methodsS3_1.8.2
## 
## loaded via a namespace (and not attached):
##  [1] tidyselect_1.2.0        farver_2.1.1            fastmap_1.1.1          
##  [4] fontquiver_0.2.1        promises_1.2.1          digest_0.6.34          
##  [7] timechange_0.2.0        mime_0.12               lifecycle_1.0.4        
## [10] Deriv_4.1.3             gfonts_0.2.0            ellipsis_0.3.2         
## [13] magrittr_2.0.3          compiler_4.3.0          rlang_1.1.3            
## [16] sass_0.4.8              utf8_1.2.4              yaml_2.3.8             
## [19] data.table_1.14.10      knitr_1.45              ggsignif_0.6.4         
## [22] labeling_0.4.3          askpass_1.2.0           bit_4.0.5              
## [25] curl_5.2.0              xml2_1.3.6              abind_1.4-5            
## [28] httpcode_0.3.0          withr_3.0.0             grid_4.3.0             
## [31] fansi_1.0.6             gdtools_0.3.5           xtable_1.8-4           
## [34] colorspace_2.1-0        scales_1.3.0            MASS_7.3-60.0.1        
## [37] crul_1.4.0              cli_3.6.2               rmarkdown_2.25         
## [40] crayon_1.5.2            ragg_1.2.7              generics_0.1.3         
## [43] rstudioapi_0.15.0       tzdb_0.4.0              cachem_1.0.8           
## [46] parallel_4.3.0          vctrs_0.6.5             Matrix_1.6-5           
## [49] carData_3.0-5           jsonlite_1.8.8          fontBitstreamVera_0.1.1
## [52] car_3.1-2               hms_1.1.3               bit64_4.0.5            
## [55] rstatix_0.7.2           systemfonts_1.0.5       jquerylib_0.1.4        
## [58] glue_1.7.0              cowplot_1.1.2           stringi_1.8.3          
## [61] gtable_0.3.4            later_1.3.2             munsell_0.5.0          
## [64] pillar_1.9.0            htmltools_0.5.7         openssl_2.1.1          
## [67] R6_2.5.1                microbenchmark_1.4.10   textshaping_0.3.7      
## [70] vroom_1.6.5             evaluate_0.23           shiny_1.8.0            
## [73] lattice_0.22-5          highr_0.10              backports_1.4.1        
## [76] broom_1.0.5             fontLiberation_0.1.0    httpuv_1.6.13          
## [79] bslib_0.6.1             Rcpp_1.0.12             zip_2.3.0              
## [82] uuid_1.2-0              gridExtra_2.3           officer_0.6.3          
## [85] xfun_0.41               pkgconfig_2.0.3
```

---

END OF SCRIPT
