# Supplementary material for: Exploring early Acheulian technological decision-making: A controlled experimental approach to raw material selection for percussive artifacts in Melka Wakena, Ethiopia
Source: PLoS One. 2025 Jan 9;20(1):e0314039. doi: 10.1371/journal.pone.0314039 (PMC11717217; doi:10.1371/journal.pone.0314039)
Supplement: S4 File — (PDF) [file pone.0314039.s004.pdf]

Table S4

Absolute and relative frequencies of some artifact categories (per raw materials) recovered from excavation at MW2 and MW5 localities.

Raw material	MW2-L3				MW5-L1&L2			
	Cores	***LCTs****	***Hammerstones***	***Modified items****	***Cores***	***LCTs****	***Hammerstones***	***Modified items****
Glassy ign.	**82** *(31.7%)*	**10** *(83.3%)*	**14** *(30.4%)*	**6** *(12.7%)*	**23** *(38.9%)*	**105** *(83.3%)*	-	**9** *(27.3%)*
Ignimbrite	**129** *(49.8%)*	**2** *(16.7%)*	**2** *(4.3%)*	**28** *(59.6%)*	**22** *(37.3%)*	**21** *(16.7%)*	**6** *(42.9%)*	**23** *(69.7%)*
Pumiceous ign.	**30** *(11.6%)*	**-**	-	**6** *(12.7%)*	**2** *(3.4%)*	-	-	**8** *(24.2%)*
Basalt	**14** *(5.4%)*	**-**	**24** *(52.2%)*	**1** *(2.1%)*	**12** *(20.3%)*	-	**7** *(50.0%)*	**14** *(42.4%)*
Scoria	**4** *(1.5%)*	**-**	**6** *(13.0%)*	**3** *(6.4%)*	-	-	**1** *(7.1%)*	-
Other volcanic	**-**	**-**	-	**3** *(6.4%)*	-	-	-	-
Total	**259**	**12**	**46**	**47**	**59**	**126**	**14**	**33**

*LCTs (Large Cutting Tools) include picks, handaxes, cleavers and large scrapers.

**Modified items are cobbles/pebbles that manifest surface modifications resembling flake removal scars but cannot be confidently categorized as either cores or percussive materials.

Note: MW2-L3 assemblages are dated to ca. 1.6 Ma and MW5-L1&L2 are ca. 1.35 Ma old (Hovers et al. 2021).
